# Enhancing the Therapeutic Potential of Peptide Antibiotics Using Bacteriophage Mimicry Strategies

**DOI:** 10.1002/advs.202411753

**Published:** 2024-11-25

**Authors:** Hongping Wan, Xinyi Zhong, Shinong Yang, Jiarong Deng, Xu Song, Yong Liu, Yuanfeng Li, Zhongqiong Yin, Xinghong Zhao

**Affiliations:** ^1^ Center for Sustainable Antimicrobials Department of Pharmacy, College of Veterinary Medicine Sichuan Agricultural University Chengdu 611130 China; ^2^ Center for Infectious Diseases Control (CIDC) Sichuan Agricultural University Chengdu 611130 China; ^3^ State Key Laboratory of Medicinal Chemical Biology Nankai University Tianjin 300071 China; ^4^ Translational Medicine Laboratory The First Affiliated Hospital of Wenzhou Medical University Wenzhou Zhejiang 325035 China

**Keywords:** antibiotic delivery, antibiotic resistance, antimicrobial peptide, bacteriophage mimicry, nanodelivery system

## Abstract

The rise of antibiotic resistance, coupled with a dwindling antibiotic pipeline, presents a significant threat to public health. Consequently, there is an urgent need for novel therapeutics targeting antibiotic‐resistant pathogens. Nisin, a promising peptide antibiotic, exhibits potent bactericidal activity through a mechanism distinct from that of clinically used antibiotics. However, its cationic nature leads to hemolysis and cytotoxicity, which has limited its clinical application. Here, nanodelivery systems have been developed by mimicking the mechanisms bacteriophages use to deliver their genomes to host bacteria. These systems utilize bacteriophage receptor‐binding proteins conjugated to loading modules, enabling efficient targeting of bacterial pathogens. Peptide antibiotics are loaded via dynamic covalent bonds, allowing for infection microenvironment‐responsive payload release. These nanodelivery systems demonstrate remarkable specificity against target pathogens and effectively localize to bacteria‐infected lungs in vivo. Notably, they significantly reduce the acute toxicity of nisin, rendering it suitable for intravenous administration. Additionally, these bacteriophage‐mimicking nanomedicines exhibit excellent therapeutic efficacy in a mouse model of MRSA‐induced pneumonia. The facile synthesis, potent antimicrobial performance, and favorable biocompatibility of these nanomedicines highlight their potential as alternative therapeutics for combating antibiotic‐resistant pathogens. This study underscores the effectiveness of bacteriophage mimicry as a strategy for transforming peptide antibiotics into viable therapeutics.

## Graphical abstract

Wan et al. present an innovative peptide antibiotic delivery approach that replicates the targeted genome delivery mechanisms of bacteriophages. This strategy substantially reduces nisin's acute toxicity, permitting safe intravenous administration and enhancing its therapeutic efficacy in a mouse model of MRSA‐induced pneumonia. This study underscores the effectiveness of bacteriophage mimicry as a strategy for transforming peptide antibiotics into viable therapeutics.

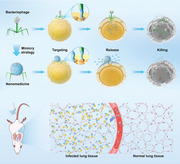

## 1 Introduction

The rise of antibiotic resistance, coupled with a declining antibiotic pipeline, poses a substantial threat to global public health.^[^ ^1^, ^2^, ^3^ ^]^ The ESKAPE pathogens‐*Enterococcus faecium*, *Staphylococcus aureus*, *Klebsiella pneumoniae*, *Acinetobacter baumannii*, *Pseudomonas aeruginosa*, and *Enterobacter* species‐have been identified as critical antibiotic‐resistant bacteria for which novel therapeutic strategies are desperately needed.^[^ ^3^, ^4^, ^5^, ^6^, ^7^ ^]^ Developing new first‐in‐class antibiotics with novel modes of action remains the most effective approach to combat these resistant pathogens.

Peptide antibiotics represent a diverse and valuable source of antimicrobial agents, including clinically approved vancomycin,^[^ ^8^ ^]^ the food preservative nisin,^[^ ^9^ ^]^ the newly discovered brevicidine,^[^ ^10^, ^11^ ^]^ and many others.^[^ ^8^, ^12^, ^13^, ^14^, ^15^, ^16^, ^17^, ^18^, ^19^ ^]^ Nisin, a ribosomally synthesized and post‐translationally modified cationic lanthipeptide, was discovered in 1928, the same year Alexander Fleming identified penicillin.^[^ ^20^ ^]^ This peptide antibiotic exhibits potent antimicrobial activity against bacterial pathogens, including *E. faecium* and *S. aureus*, both members of the ESKAPE pathogens.^[^ ^21^, ^22^ ^]^ Nisin exerts its bactericidal effects by targeting the pyrophosphate group of lipid II, an essential and conserved moiety, leading to the formation of nisin‐lipid II hybrid pores in the bacterial membrane and the inhibition of cell wall synthesis through lipid II sequestration.^[^ ^23^, ^24^, ^25^ ^]^

Remarkably, no nisin‐resistant genes have been reported in foodborne pathogens after more than half a century of its use as a food preservative worldwide, a testament to its novel mode of action. Moreover, nisin's mechanism of action is distinct from that of any clinically used antibiotic, highlighting its potential as an ideal first‐in‐class candidate for combating antibiotic‐resistant pathogens. However, its cationic nature, which leads to hemolysis and cytotoxicity, has impeded its development as a therapeutic antibiotic. Consequently, innovative strategies to transform nisin into a viable intravenous antibiotic are urgently needed.

**Figure 1:**
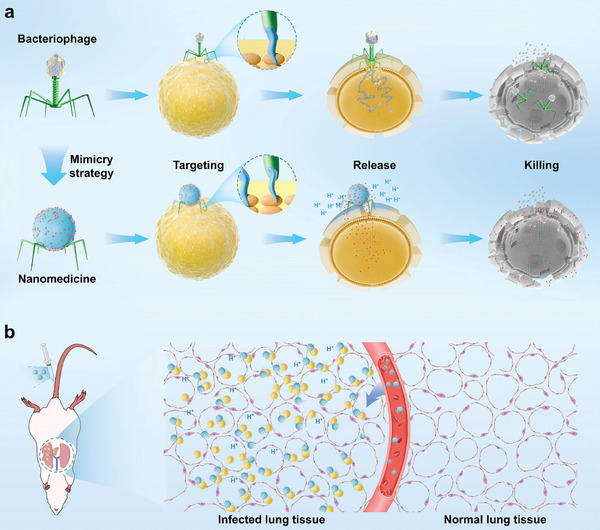
Schematic illustrations of the creation of bacteriophage‐mimicking nanomedicines. a), Bacteriophage‐mimicking nanomedicines were developed to emulate the process by which bacteriophages deliver their genome to host bacteria. These nanomedicines possess two key features: 1) the ability to specifically and effectively capture target bacterial pathogens through RBPs, and 2) the precise release of peptide antibiotics at the infection site, driven by the infection microenvironment. b), In a mouse model of MRSA‐induced pneumonia, these nanomedicines effectively accumulated in the infected lung tissues, enabling targeted delivery of the microenvironment‐responsive peptide antibiotic payload.

Bacteriophages are viruses that specifically infect bacteria.^[^ ^26^ ^]^ The exquisite specificity of bacteriophage‐host interactions is primarily mediated by bacteriophage receptor‐binding proteins (RBPs).^[^ ^27^, ^28^ ^]^ To successfully infect their bacterial hosts, bacteriophages utilize RBPs to efficiently capture specific hosts and then precisely deliver their genome into the host bacteria via specialized tail machinery.^[^ ^29^, ^30^ ^]^ Inspired by the mechanism of bacteriophages delivering their genome to bacterial hosts, we propose that bacteriophage mimicry strategies could be an effective approach for enhancing the therapeutic potential of nisin (**Figure** **1**a).

For bacteriophage mimicry delivery systems to accurately and effectively deliver nisin in the treatment of bacterial infections, they must possess two fundamental properties: 1) the ability to capture target bacterial pathogens with exquisite specificity and efficacy, and 2) the capacity to release peptide antibiotics precisely at the site of infection (Figure 1a).

Recently, we demonstrated that RBPs are highly effective in directing nanoparticles to specific bacterial pathogens.^[^ ^31^ ^]^ This efficiency is anticipated, as studies have shown that RBPs possess antibody‐like specificity and affinity for host bacteria.^[^ ^27^, ^30^, ^32^ ^]^ These findings suggest that the first fundamental property of bacteriophage mimicry delivery systems can be achieved by employing RBPs as targeting modules.

The pH at the site of bacterial infections (5.0–6.0) is significantly lower than the physiological pH of 7.4, providing an opportunity to develop infection microenvironment‐responsive delivery systems.^[^ ^33^, ^34^ ^]^ The primary amino group of peptide antibiotics can react with aldehyde groups to form imine bonds.^[^ ^35^, ^36^ ^]^ These imine bonds are stable at physiological pH but highly sensitive to acidic conditions.^[^ ^33^, ^37^ ^]^ By utilizing imine bond‐based dynamic connections, we have successfully developed dynamic covalent nano‐networks and amphiphilic assemblies that can overcome antibiotic resistance in peptide antibiotics.^[^ ^38^, ^39^ ^]^ In addition, these infection microenvironment‐responsive delivery systems can minimize the interaction of peptide antibiotics with normal cells and tissues, thereby significantly reducing their cytotoxicity and hemolytic activity.^[^ ^38^ ^]^ Consequently, the second fundamental property of bacteriophage mimicry delivery systems can be achieved by employing dynamic covalent connections.

In this study, three distinct bacteriophage mimicry delivery systems were developed to enhance the therapeutic potential of nisin against methicillin‐resistant *S. aureus* (MRSA) infections. First, dynamic covalent amphiphilic assemblies of nisin with natural aldehydes were engineered, utilizing a broad‐host‐range *S. aureus*‐specific RBP, RBP_sb1_, as the targeting module to achieve precise and effective capture of bacterial pathogens. Second, dynamic covalent nano‐networks composed of nisin, 2‐formylphenylboronic acid, and natural polyphenols were synthesized and conjugated with RBP_sb1_ via maleimide‐mediated bioconjugation. Third, nisin‐loaded, biocompatible urchin‐like porous silica nanoparticles (UPSNs) were created using dynamic covalent connections and further functionalized with RBP_sb1_. All three bacteriophage mimicry delivery systems significantly reduced the cytotoxicity, hemolytic activity, and acute toxicity of nisin, rendering it suitable for intravenous administration. In addition, these systems markedly improved the therapeutic efficacy of nisin in a mouse MRSA pneumonia model (Figure 1b). This study demonstrates that bacteriophage mimicry strategies are effective in transforming peptide antibiotics into viable therapeutics for combating antibiotic‐resistant pathogens.

## 2 Results

### 2.1 Biosynthesis of the *S. aureus*‐Targeting Module

**Figure 2:**
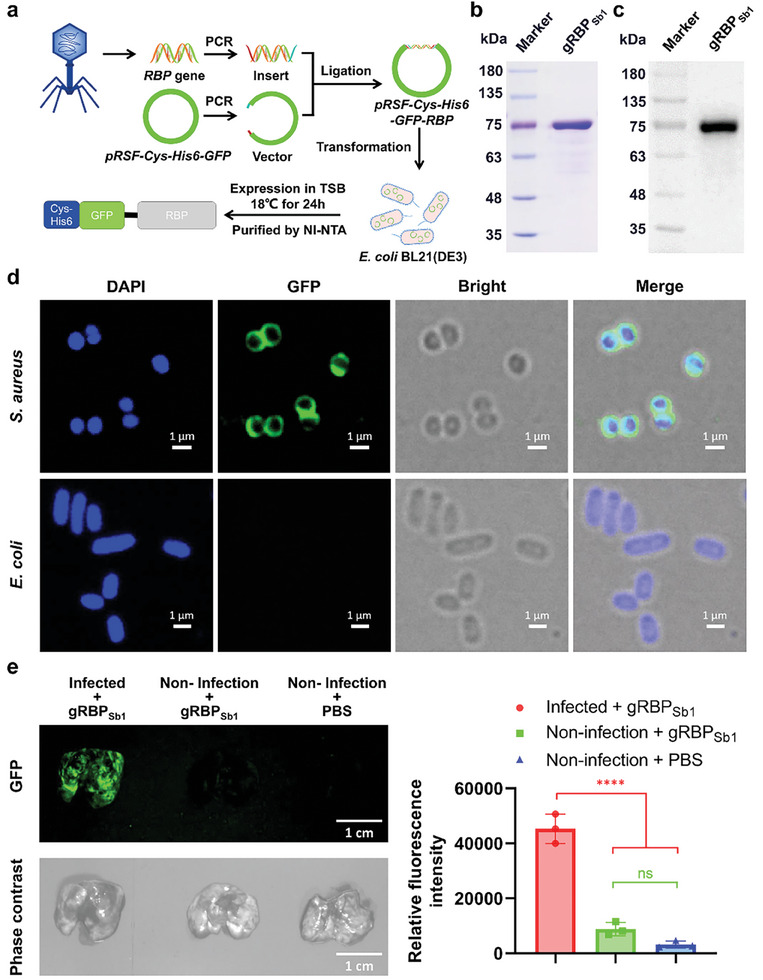
Heterologous expressed gRBP_sb1_ selectively binds to cultured pathogenic bacteria and accumulates efficiently at the site of infections. a), Schematic representation of the heterologous expression of gRBP_sb1_. b) SDS‐PAGE images and c) anti‐His6 western blot of the heterologously expressed gRBP_sb1_. Experiments were repeated three times with similar results. d), Confocal laser scanning microscopy images of MRSA and *E. coli* after incubation with gRBP_sb1_ (green). Pathogenic bacteria are visualized under phase contrast, and bacterial nucleoids are stained with DAPI (blue). Experiments were repeated three times with similar results. e), Time‐gated fluorescence image of gRBP_sb1_ in lungs harvested from mice 30 min after intravenous injection. MRSA (6 × 10⁹ c.f.u. per mouse) was used to induce lung infection by intratracheal inoculation. At 24 h post‐infection, gRBP_sb1_ (0.5 mg per mouse) was injected intravenously and allowed to circulate for 30 min. Lungs were then harvested for time‐gated fluorescence imaging using the FUSION FX7 EDGE Imaging System. Mice without MRSA infection were treated with the same dose of gRBP_sb1_ or an equivalent volume of PBS as controls. Data are presented as mean ± standard deviation (n = 3 biological replicates). Statistical significance was assessed using one‐way ANOVA followed by Tukey's multiple comparisons test. ns, no significance; ^****^ *p* < 0.0001.

To develop bacteriophage mimicry delivery systems that target bacterial pathogens with high specificity and efficacy, we utilized the RBPs from bacteriophages.^[^ ^28^, ^29^ ^]^ To achieve broad‐spectrum targeting of *S. aureus*, we biosynthesized the RBP from the staphylococcal bacteriophage Sb‐1 (RBP_sb1_), a crucial component in therapeutic phage preparations.^[^ ^40^ ^]^ Specifically, the gene encoding RBP_sb1_ (Table S1, Supporting Information) was cloned into a plasmid encoding Cys‐6xHis‐GFP,^[^ ^31^ ^]^ resulting in the production of Cys‐6xHis‐GFP‐RBP_sb1_ (gRBP_sb1_; Table S2, Supporting Information) in *E. coli* BL21(DE3) (**Figure** **2**a). Following verification by sequencing, the plasmid was transformed into *E. coli* BL21(DE3) to express gRBP_sb1_. After purification, the gRBP_sb1_ was analyzed by sodium dodecyl sulfate‐polyacrylamide gel electrophoresis (SDS‐PAGE) and Western blotting, confirming the expected size of 79 kDa and high purity (Figure 2b,c).

Next, the *S. aureus*‐specific targeting capability of gRBP_sb1_ was assessed using fluorescence microscopy and confocal laser scanning microscopy (CLSM). Following incubation respectively with various bacterial strains (Table S3, Supporting Information), a high‐intensity green fluorescence signal was observed for all twelve *S. aureus* strains (Figure 2d; Figure S1, Supporting Information), whereas no fluorescence signals were detected for the five non‐target strains (Figure S2, Supporting Information). These results demonstrate that gRBP_sb1_ is a broad‐host‐range, *S. aureus*‐specific targeting molecule. This finding is consistent with previous studies indicating that RBPs have a broader targeting spectrum against bacterial strains compared to fully assembled bacteriophages.^[^ ^41^, ^42^ ^]^

After confirming that gRBP_sb1_ targets *S. aureus* with high specificity and efficacy in *vitro*, we evaluated its targeting capability in *vivo*. For this purpose, gRBP_sb1_ was administered to mice with MRSA pneumonia. Following a 30‐min circulation period, the lungs were collected for time‐gated fluorescence imaging analysis. The images revealed that gRBP_sb1_ accumulated significantly in the lungs of MRSA‐infected mice (Figure 2e), whereas no accumulation was observed in non‐infected mice (Figure 2e). These results demonstrate that gRBP_sb1_ possesses effective in vivo *S. aureus*‐specific targeting ability and could serve as a targeting module in bacteriophage mimicry delivery systems.

### 2.2 Creation and Characterization of the Bacteriophage‐mimicking Nanomedicines

To achieve high loading efficacy and infection microenvironment‐responsive release of nisin, we developed three distinct loading modules utilizing advanced nanotechnologies for peptide antibiotics: 1) dynamic covalent amphiphile assemblies of nisin and natural aldehydes, 2) dynamic covalent nano‐networks composed of nisin, polyphenols, and a cross‐linker 4‐acetylbenzeneboronic acid, and 3) dynamic covalent Nisin@UPSNs.

**Figure 3:**
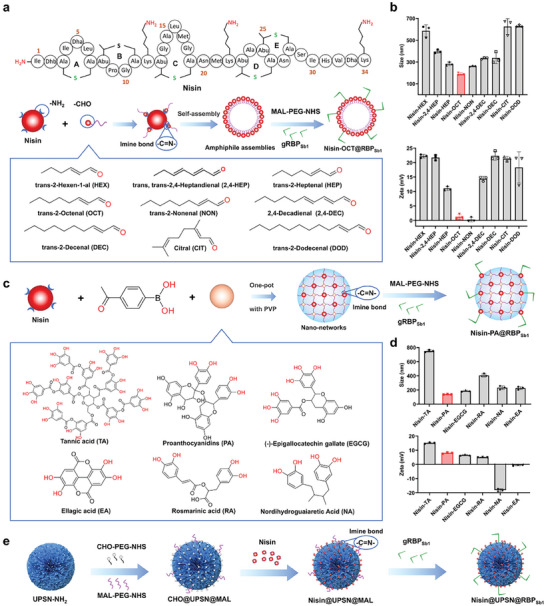
Design and synthesis of bacteriophage‐mimicking nanomedicines. a), Schematic illustration of the synthesis and self‐assembly of the designed amphiphiles, including the chemical structures of the aldehydes used in this study. The yielded dynamic covalent amphiphile assemblies were further conjugated with gRBP_Sb1_ to produce the type I bacteriophage‐mimicking nanomedicine, Nisin‐OCT@RBP_Sb1_. b), Size distribution and zeta potential of the various amphiphile assemblies measured by dynamic laser scattering. Data are presented as mean ± standard deviation (n = 3 independent experiments) c), Diagram depicting the preparation of the iminoboronate‐stabilized nano‐networks, including the chemical structures of 4‐acetylbenzeneboronic acid and the polyphenols used. The formed dynamic covalent nano‐networks were further conjugated with gRBP_Sb1_ to produce the type II bacteriophage‐mimicking nanomedicine, Nisin‐PA@RBP_Sb1_. d), Size distribution and zeta potential of the various nano‐networks measured via dynamic laser scattering. Data are presented as mean ± standard deviation (n = 3 independent experiments). e), Preparation route for the type III bacteriophage‐mimicking nanomedicine, Nisin@UPSN@RBP_Sb1_.

First, dynamic covalent amphiphile assemblies of nisin and natural aldehydes were synthesized using a previously described method.^[^ ^38^ ^]^ To adapt the formulation from polymyxin B to nisin, nine natural aldehydes were selected to create a variety of dynamic covalent amphiphile assemblies (**Figure** **3**a). The results indicate that the dynamic covalent amphiphile assembly (Nisin‐OCT), with a hydrodynamic diameter of 189 nm, was synthesized using trans‐2‐octenal (OCT) and nisin (Figure 3b). Due to its favorable hydrodynamic size, Nisin‐OCT was chosen for conjugation with gRBP_Sb1_ via a maleimide‐thiol reaction, resulting in type I bacteriophage‐mimicking nanomedicines (Nisin‐OCT@RBP_Sb1_) (Figure 3a).

Second, a straightforward synthetic method was employed to create dynamic covalent nano‐networks,^[^ ^39^ ^]^ which consisted of peptide antibiotics, polyphenols, and a cross‐linker 4‐acetylbenzeneboronic acid. To adapt the formulation from polymyxins to nisin, six natural polyphenols were selected to produce various dynamic covalent nano‐networks (Figure 3c). Using the formulation of nisin, proanthocyanidins, and the cross‐linker 4‐acetylbenzeneboronic acid, nisin‐loaded nano‐networks (nisin‐PA) with a hydrodynamic diameter of 139 nm were synthesized (Figure 3d). Subsequently, a heterobifunctional maleimide‐polyethylene glycol‐N‐hydroxysuccinimide (MW = 5000 Da, MAL‐PEG‐NHS) was conjugated to the nisin‐PA via an amine‐NHS reaction, and further linked to gRBP_Sb1_ through a MAL‐thiol reaction, resulting in type II bacteriophage‐mimicking nanomedicines (Nisin‐PA@RBP_Sb1_) (Figure 3c).

Third, UPSNs were chosen as the loading module due to their high loading capacity and favorable biocompatibility.^[^ ^43^, ^44^, ^45^ ^]^ They were synthesized using a previously described method.^[^ ^31^, ^46^ ^]^ Following amination, heterobifunctional aldehyde‐polyethylene glycol‐N‐hydroxysuccinimide (MW = 1000 Da, CHO‐PEG‐NHS) and MAL‐PEG‐NHS (MW = 5000Da) were conjugated to UPSNs at a molar ratio of 100:1 via an amine‐NHS reaction. Nisin was then loaded onto the modified UPSNs through dynamic covalent bonding via an amine‐CHO reaction (Figure 3e). Finally, gRBP_Sb1_ was conjugated to the nisin‐loaded UPSNs through a MAL‐thiol reaction, resulting in type III bacteriophage‐mimicking nanomedicines (Nisin@UPSN@RBP_Sb1_) (Figure 3e).

**Figure 4:**
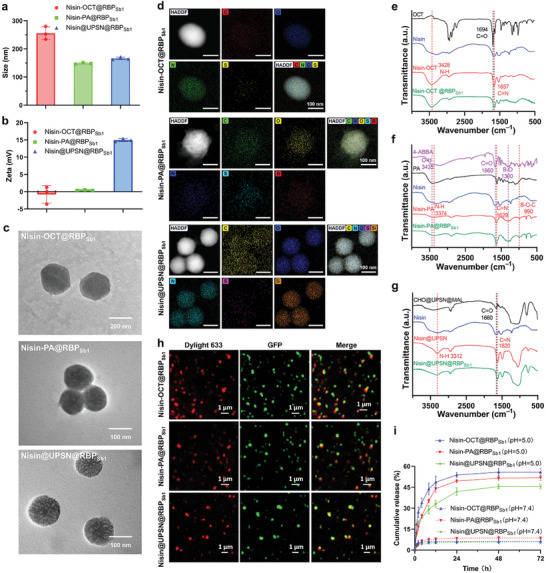
Characterization of bacteriophage‐mimicking nanomedicines. a), Average hydrodynamic size of Nisin‐OCT@RBP_Sb1_, Nisin‐PA@RBP_Sb1_, and Nisin@UPSN@RBP_Sb1_, measured by dynamic light scattering. Data are presented as mean ± standard deviation (n = 3 independent experiments). b), Surface zeta‐potential of Nisin‐OCT@RBP_Sb1_, Nisin‐PA@RBP_Sb1_, and Nisin@UPSN@RBP_Sb1_ in ultrapure water (1 mg mL^−1^). Data are presented as mean ± standard deviation (n = 3 independent experiments). c), Transmission electron microscope images of Nisin‐OCT@RBP_Sb1_, Nisin‐PA@RBP_Sb1_, and Nisin@UPSN@RBP_Sb1_. d), EDS elemental mapping of Nisin‐OCT@RBP_Sb1_, Nisin‐PA@RBP_Sb1_, and Nisin@UPSN@RBP_Sb1_. e—g), FT‐IR spectra of Nisin‐OCT@RBP_Sb1_ (e), Nisin‐PA@RBP_Sb1_ (f), and Nisin@UPSN@RBP_Sb1_ (g). h), Confocal laser scanning microscopy images of Nisin‐OCT@RBP_Sb1_, Nisin‐PA@RBP_Sb1_, and Nisin@UPSN@RBP_Sb1_ in which the loading module was labeled with DyLight 633 (red) and the targeting devices were fused with GFP (green). i), Release profiles of nisin payload from the nanomedicines in PBS at pH = 5.0 or pH = 7.4, at 37 °C. Data are presented as mean ± standard deviation (n = 3 independent experiments).

Following preparation, the generated bacteriophage‐mimicking nanomedicines were characterized. Dynamic light scattering (DLS) was first used to measure the hydrodynamic diameters and zeta potentials of the nanomedicines (**Figure** **4**a,b). The results revealed that Nisin‐OCT@RBP_Sb1_, Nisin‐PA@RBP_Sb1_, and Nisin@UPSN@RBP_Sb1_ had average hydrodynamic diameters of 255.4 ± 23.0 nm, 148.3 ± 2.8 nm, and 165.9 ± 4.8 nm (Table S4; Figure S3, Supporting Information), respectively, which were slightly larger than those of the gRBP_Sb1_‐absent nanoparticles, suggesting successful conjugation of the targeting module. In addition, Nisin‐OCT@RBP_Sb1_, Nisin‐PA@RBP_Sb1_, and Nisin@UPSN@RBP_Sb1_ exhibited average zeta potentials of ‐0.8 ± 2.5 mV, 0.4 ± 0.1 mV, and 14.9 ± 0.5 mV (Table S4, Supporting Information), respectively, significantly lower than those of the gRBP_Sb1_‐absent nanoparticles. This reduction in zeta potential further suggests successful conjugation of negatively charged gRBP_Sb1_ (in ultrapure water).

Next, transmission electron microscopy (TEM) was used to analyze the morphologies of the bacteriophage‐mimicking nanomedicines. As shown in Figure 4c, uniform structures of Nisin‐OCT@RBP_Sb1_, Nisin‐PA@RBP_Sb1_, and Nisin@UPSN@RBP_Sb1_ were successfully observed. Similar morphologies were noted for the gRBP_Sb1_‐absent nanoparticles (Figure S4, Supporting Information). Moreover, energy dispersive spectroscopy (EDS) elemental mapping confirmed the uniform distribution of the representative elements within the respective bacteriophage‐mimicking nanomedicines (Figure 4d).

To verify the formation of dynamic covalent bonds (imine bonds) in Nisin‐OCT@RBP_Sb1_, Nisin‐PA@RBP_Sb1_, and Nisin@UPSN@RBP_Sb1_, Fourier transform infrared spectroscopy (FT‐IR) analysis was performed. In the Nisin‐OCT@RBP_Sb1_ system (Figure 4e), the broad absorption peak and two sharp peaks between 3000 and 3300 cm^−1^ (corresponding to the primary amine group of nisin) as well as the absorption peak around 1694 cm^−1^ (C═O stretching vibration) disappeared after the reaction. A new peak appeared around 1657 cm^−1^ (C═N stretching vibration), indicating the formation of imine bonds in Nisin‐OCT@RBP^Sb1^. Additionally, the N─H bond of the amide group remained intact, suggesting that the reaction did not affect the nisin structure. Similar results were also observed for Nisin‐PA@RBP_Sb1_ and Nisin@UPSN@RBP_Sb1_, indicating imine bond formation in these nanomedicines (Figure 4f,g). Furthermore, the stretching vibration of the B‐O bond in 4‐acetylbenzeneboronic acid, observed at 1300 cm^−1^, disappeared after the reaction (Figure 4g). Conversely, new absorption peaks around 990 cm^−1^ (B‐O‐C stretching vibration) appeared, indicating the formation of boronate bonds in Nisin‐PA@RBP_Sb1_. These results collectively confirm that dynamic covalent bonds (imine bonds) were successfully formed during the fabrication of these bacteriophage‐mimicking nanomedicines.

After verifying the correct composition of the loading modules, the presence of gRBP_Sb1_ in the bacteriophage‐mimicking nanomedicines was confirmed using confocal laser scanning microscopy (CLSM) (Figure 4h). For this analysis, the nisin‐loaded modules were labeled with DyLight 633, a red fluorescence dye. Subsequently, the amount of gRBP_Sb1_ on the bacteriophage‐mimicking nanomedicines was quantified using a fluorescence‐based approach, employing a standard curve generated from the fluorescence intensity of a concentration series of gRBP_Sb1_ (Figure S5, Supporting Information). The results show that Nisin‐OCT@RBP_Sb1_, Nisin‐PA@RBP_Sb1_, and Nisin@UPSN@RBP_Sb1_ contained targeting module amounts of 1.19 ± 0.13 nmol mg^−1^, 1.32 ± 0.12 nmol mg^−1^, and 0.86 ± 0.08 nmol mg^−1^, respectively (Table S5, Supporting Information). In addition, high‐performance liquid chromatography (HPLC) analysis revealed that Nisin‐OCT@RBP_Sb1_, Nisin‐PA@RBP_Sb1_, and Nisin@UPSN@RBP_Sb1_ had nisin loading efficiencies of 76.8%, 82.8%, and 63.0%, respectively (Table S6, Supporting Information).

Finally, the nisin release profiles of Nisin‐OCT@RBP_Sb1_, Nisin‐PA@RBP_Sb1_, and Nisin@UPSN@RBP_Sb1_ were evaluated under physiological (PBS, pH 7.4) and acidic conditions (PBS, pH 5.0) to simulate severe infection environments. As shown in Figure 4i, the nanomedicines exhibited negligible nisin release at physiological pH. In contrast, under acidic conditions (PBS, pH 5.0), Nisin‐OCT@RBP_Sb1_, Nisin‐PA@RBP_Sb1_, and Nisin@UPSN@RBP_Sb1_ released nisin more rapidly, with release percentages of 55.8%, 52.0%, and 45.5%, respectively. This increased release rate was attributed to the dynamic covalent bonds between nisin and the nanodelivery systems. Similar results were observed for nanoparticles without gRBP_Sb1_ (Figure S6, Supporting Information). These findings demonstrate that the bacteriophage‐mimicking nanomedicines‐Nisin‐OCT@RBP_Sb1_, Nisin‐PA@RBP_Sb1_, and Nisin@UPSN@RBPSb1‐exhibit a favorable infection microenvironment‐responsive nisin release property, and their *S. aureus* targeting efficacy could be assessed.

### 2.3 Bacteriophage‐Mimicking Nanomedicines Target *S. aureus* Efficiently

**Figure 5:**
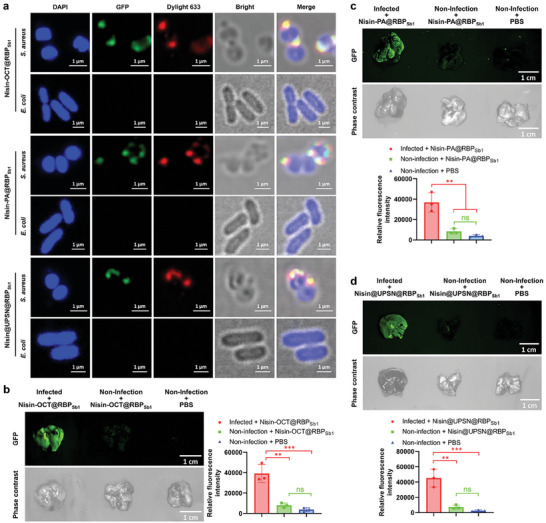
Bacteriophage‐mimicking nanomedicines selectively target pathogenic bacteria and localize to infected lungs in vivo. a), Confocal laser scanning microscopy images of MRSA and *E. coli* after incubation with Nisin‐OCT@RBP_Sb1_, Nisin‐PA@RBP_Sb1_, or Nisin@UPSN@RBP_Sb1_ in which the loading module was labeled with DyLight 633 (red) and the targeting devices were fused with GFP (green). Pathogenic bacteria are visualized under phase contrast, and bacterial nucleoid was stained with DAPI (blue). Experiment was repeated three times with similar results. b—d), Time‐gated fluorescence images of Nisin‐OCT@RBP_Sb1_ (b), Nisin‐PA@RBP_Sb1_ (c), and Nisin@UPSN@RBP_Sb1_ (d), in which RBP_sb1_ was fused with GFP (green), in lungs harvested from mice 30 min after intravenous injection. *S. aureus*‐induced lung infection was generated by intratracheal inoculation of MRSA (6 × 10⁹ c.f.u. per mouse). At 24 h post‐infection, Nisin‐OCT@RBP_Sb1_, Nisin‐PA@RBP_Sb1_, and Nisin@UPSN@RBP_Sb1_ were intravenously injected, respectively, and allowed to circulate for 30 min. Lungs were then harvested for time‐gated fluorescence imaging using a FUSION FX7 EDGE Imaging System. Mice without MRSA infection were treated with the same dose (4 mg per mouse) of each nanomedicine or with PBS as controls. Data are presented as mean ± standard deviation (n = 3 biological replicates). Statistical significance was assessed using one‐way ANOVA followed by Tukey's multiple comparisons test. ns, no significance; ^**^ *p* < 0.01; ^***^ *p* < 0.001.

After confirming the infection microenvironment‐responsive nisin release property, we assessed the capability of the bacteriophage‐mimicking nanomedicines to capture target bacterial pathogens. DAPI‐stained bacterial pathogens were treated with DyLight‐633‐labeled bacteriophage‐mimicking nanomedicines and subsequently analyzed using CLSM. As shown in **Figure** **5**a, all three nanomedicines efficiently bound to the target pathogen, MRSA, while none interacted with the non‐target pathogen, *E. coli*. These results indicate that the bacteriophage‐mimicking nanomedicines can capture *S. aureus* with high specificity and efficacy, suggesting their potential for targeting the site of *S. aureus* infections in *vivo*.

To evaluate the in vivo targeting efficacy of the bacteriophage‐mimicking nanomedicines, we performed time‐gated fluorescence imaging analysis on lung tissues from infected mice after administering the nanomedicines. As shown in Figure 5b–d, all three nanomedicines were significantly accumulated in the lungs of MRSA‐infected mice. In contrast, the nanomedicines showed no significant accumulation in the lungs of non‐infected mice (Figure 5b–d). These results indicate that the bacteriophage mimicking nanomedicines exhibit a bacteriophage‐like bacterial targeting profile, which is advantageous for directing the infection microenvironment‐responsive loading modules specifically to the site of infection with exquisite specificity and efficacy.

### 2.4 Bacteriophage Mimicry Strategy Makes Nisin an Intravenous Administrable Antibiotic

**Figure 6:**
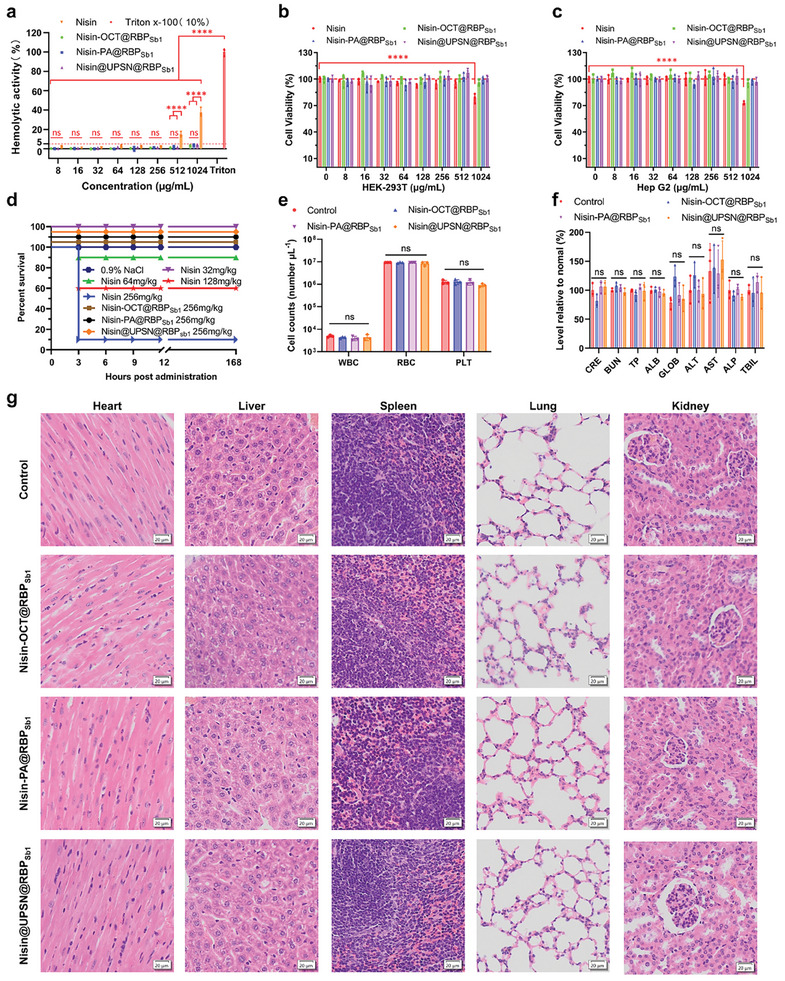
Biosafety assessment of Nisin‐OCT@RBP_Sb1_, Nisin‐PA@RBP_Sb1_, and Nisin@UPSN@RBP_Sb1_. a), Hemolytic Activity: The hemolytic activity of free nisin, Nisin‐OCT@RBP_Sb1_, Nisin‐PA@RBP_Sb1_, and Nisin@UPSN@RBP_Sb1_. Data are presented as mean ± standard deviation (n = 3 biological replicates). Statistical significance was assessed using one‐way ANOVA followed by Tukey's multiple comparisons test. ns, no significance; ^****^ *p* < 0.001. b,c), Cell Viability: The viability of HEK‐293T (b) and Hep G2 (c) cells after treatment with free nisin, Nisin‐OCT@RBP_Sb1_, Nisin‐PA@RBP_Sb1_, or Nisin@UPSN@RBP_Sb1_ at concentrations ranging from 8 to 1024 µg mL^−1^ for 24 h. Data are presented as mean ± standard deviation (n = 3 biological replicates). Statistical significance was assessed using one‐way ANOVA followed by Tukey's multiple comparisons test. ns, no significance; ^****^ *p* < 0.001. d), Acute toxicity: Mice were treated with free nisin, Nisin‐OCT@RBP_Sb1_, Nisin‐PA@RBP_Sb1_, or Nisin@UPSN@RBP_Sb1_ at doses of 32, 64, 128, and 256 mg kg^−1^, respectively. Mortality was monitored for 7 days. e), Blood Cell Counts: Counts of white blood cells (WBC), red blood cells (RBC), and platelets (PLT) were measured 7 days after administration of Nisin‐OCT@RBP_Sb1_, Nisin‐PA@RBP_Sb1_, and Nisin@UPSN@RBP_Sb1_. Data are presented as mean ± standard deviation (n = 3 biological replicates). Statistical significance was assessed using one‐way ANOVA followed by Tukey's multiple comparisons test. ns, no significance. f), Comprehensive Blood Chemistry: A comprehensive blood chemistry panel was performed 7 days after administration of Nisin‐OCT@RBP_Sb1_, Nisin‐PA@RBP_Sb1_, and Nisin@UPSN@RBP_Sb1_. Parameters include creatinine (CRE), blood urea nitrogen (BUN), total protein (TP), albumin (ALB), alanine transaminase (ALT), aspartate transaminase (AST), and alkaline phosphatase (ALP). Data are presented as mean ± standard deviation (n = 3 biological replicates). Statistical significance was assessed using one‐way ANOVA followed by Tukey's multiple comparisons test. ns, no significance. g), Histological Analysis: Haematoxylin and eosin staining of histology sections from major organs was conducted 7 days after intravenous administration of Nisin‐OCT@RBP_Sb1_, Nisin‐PA@RBP_Sb1_, and Nisin@UPSN@RBP_Sb1_. Scale bars, 20 µm. Independent experiments (n = 3 biological replicates) were performed with similar results.

The previous results confirmed the successful creation of bacteriophage‐mimicking nanomedicines. To test our hypothesis that bacteriophage mimicry could mitigate the acute toxicity of nisin and enable its intravenous administration, we conducted hemolytic activity, cytotoxicity, and in *vivo* toxicity assays on Nisin‐OCT@RBP_Sb1_, Nisin‐PA@RBP_Sb1_, and Nisin@UPSN@RBP_Sb1._^[^ ^47^, ^48^ ^]^ As shown in **Figure** **6**a, free nisin exhibited significant hemolytic activity at concentrations of 512 and 1024 µg mL^−1^ in a dose‐dependent manner. In contrast, Nisin‐OCT@RBP_Sb1_, Nisin‐PA@RBP_Sb1_, and Nisin@UPSN@RBP_Sb1_ did not induce hemolysis even at a high concentration of 1024 µg mL^−1^. We then evaluated the cytotoxicity of these nanomedicines on the hepatoblastoma cell line (Hep G2) and human embryonic kidney 293T (HEK‐293T). The results demonstrated that free nisin significantly reduced cell viability in both Hep G2 and HEK‐293T cells at 1024 µg mL^−1^ (Figure 6b,c). Conversely, Nisin‐OCT@RBP_Sb1_, Nisin‐PA@RBP_Sb1_, and Nisin@UPSN@RBP_Sb1_ had no significant effect on the viability of Hep G2 and HEK‐293T cells across a concentration range of 8 to 1024 µg mL^−1^ (Figure 6b,c). Similarly, gRBP_Sb1_‐absent nanomedicines also exhibited no hemolytic activity or cytotoxicity in the same concentration range (Figures S7 and S8, Supporting Information).

After confirming in vitro biosafety, we conducted an acute toxicity assay for Nisin‐OCT@RBP_Sb1_, Nisin‐PA@RBP_Sb1_, and Nisin@UPSN@RBP_Sb1_ in vivo. Following intravenous administration, free nisin caused severe acute toxicity, with a 50% lethal dose (LD_50_) of 137 mg kg^−1^ (Figure 6d; Table S7, Supporting Information). In contrast, Nisin‐OCT@RBP_Sb1_, Nisin‐PA@RBP_Sb1_, and Nisin@UPSN@RBP_Sb1_ exhibited no acute toxicity in mice across a dose range of 32 to 256 mg kg^−1^. These results collectively demonstrate that the intravenous toxicity of nisin is significantly reduced through bacteriophage mimicry strategies, highlighting a favorable intravenous administration profile for Nisin‐OCT@RBP_Sb1_, Nisin‐PA@RBP_Sb1_, and Nisin@UPSN@RBP_Sb1_.

To further confirm the favorable biosafety of Nisin‐OCT@RBP_Sb1_, Nisin‐PA@RBP_Sb1_, and Nisin@UPSN@RBP_Sb1_, we analyzed blood chemistry and major blood cell populations in mice 7 days post‐administration (Figure 6e,f). No significant differences were observed in these parameters between mice treated with phosphate‐buffered saline (PBS) and those treated with Nisin‐OCT@RBP_Sb1_, Nisin‐PA@RBP_Sb1_, or Nisin@UPSN@RBP_Sb1_. Additionally, body weight changes showed no significant differences between the bacteriophage‐mimicking nanomedicines‐treated groups and the PBS‐treated group (Figure S9, Supporting Information). Histopathological analysis of major organs from mice treated with Nisin‐OCT@RBP_Sb1_, Nisin‐PA@RBP_Sb1_, and Nisin@UPSN@RBP_Sb1_ revealed no visible lesions (Figure 6g). These findings collectively demonstrate that Nisin‐OCT@RBP_Sb1_, Nisin‐PA@RBP_Sb1_, and Nisin@UPSN@RBP_Sb1_ do not exhibit signs of acute toxicity, consistent with previous reports on the biosafety of phages, dynamic covalent amphiphile assemblies, dynamic covalent nano‐networks, and porous silica nanoparticles.^[^ ^38^, ^39^, ^43^, ^49^, ^50^, ^51^ ^]^

These assessments collectively demonstrate that Nisin‐OCT@RBP_Sb1_, Nisin‐PA@RBP_Sb1_, and Nisin@UPSN@RBP_Sb1_ effectively mitigate the severe acute toxicity of nisin, indicating their potential as viable intravenous therapeutics for combating antibiotic‐resistant *S. aureus*.

### 2.5 Bacteriophage‐Mimicking Nanomedicines Show Promising Therapeutic Effects

**Figure 7:**
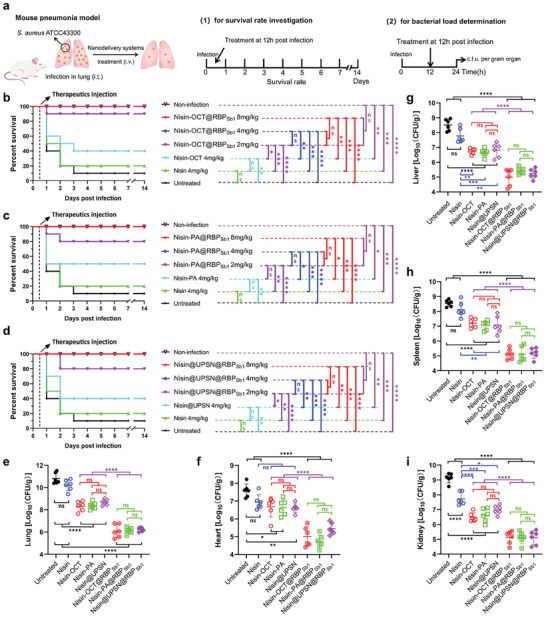
In vivo therapeutic efficacy of Nisin‐OCT@RBP_Sb1_, Nisin‐PA@RBP_Sb1_, and Nisin@UPSN@RBP_Sb1_. a), A scheme of the experimental protocol for the mouse MRSA pneumonia models. b—d), Survival rates of MRSA‐induced pneumonia mice upon treatment with a single dose of free nisin, Nisin‐OCT@RBP_Sb1_, Nisin‐PA@RBP_Sb1_, Nisin@UPSN@RBP_Sb1_, Nisin‐OCT, Nisin‐PA, Nisin@UPSN, or 0.1 M PBS via intravenous injection at 12 h post‐infection (n =   10 biological replicates). Survival analysis was performed using the Log‐rank (Mantel‐Cox) test. ns, no significance; ^*^ *p* < 0.05; ^**^ *p* < 0.01; ^***^ *p* < 0.001; ^****^ *p* < 0.0001. e—i), Treatment with Nisin‐OCT@RBP_Sb1_ (4 mg kg^−1^), Nisin‐PA@RBP_Sb1_ (4 mg kg^−1^), or Nisin@UPSN@RBP_Sb1_ significantly reduced the bacterial load in organs of MRSA‐induced mice to equivalent doses of untargeted nisin nanoparticles or of free nisin. At 24 h post‐infection, Mice (n = 6) were euthanized by cervical dislocation at 24 h post‐infection. Bacterial loads (Log10 c.f.u. per gram) in the lung (e), heart (f), liver (g), spleen (h), and kidney (i) were quantified. Data are presented as mean ± standard deviation (n = 6 biological replicates). Statistical significance was assessed using one‐way ANOVA followed by Tukey's multiple comparisons test. ns, no significance; ^*^ *p* < 0.05; ^**^ *p* < 0.01; ^***^ *p* < 0.001; ^****^ *p* < 0.0001.

Finally, the therapeutic efficacy of the bacteriophage‐mimicking nanomedicines for treating MRSA‐induced acute lung infection was evaluated. An acute lung infection model was established by intratracheal introduction of MRSA (**Figure** **7**a), which caused severe pneumonia and resulted in 90% mortality (n = 10 mice) within 12–72 h post‐infection (Figure 7b–d; Figure S10, Supporting Information). Intravenous administration of Nisin‐OCT@RBP_Sb1_, Nisin‐PA@RBP_Sb1_, and Nisin@UPSN@RBP_Sb1_ at a dose of 2 mg kg^−1^, administered 12 h post‐infection, led to recovery rates of 90%, 80%, and 80%, respectively (Figure 7b–d). In contrast, free nisin at the same dose was ineffective (Figure S10, Supporting Information). A 100% recovery rate and long‐term survival were achieved with doses of 4 or 8 mg kg^−1^ of Nisin‐OCT@RBP_Sb1_, Nisin‐PA@RBP_Sb1_, and Nisin@UPSN@RBP_Sb1_ (Figure 7b–d). However, free nisin at a dose of 4 mg kg^−1^ did not show significant therapeutic effects (Figure 7b–d). Moreover, gRBP_Sb1_‐absent nanomedicines, including Nisin‐OCT, Nisin‐PA, and Nisin@UPSN, exhibited only a 40%‐50% survival rate at 4 mg kg^−1^ (Figure 7b–d), highlighting the critical role of the targeting module in enhancing therapeutic efficacy. Notably, free nisin at a dose of 32 mg kg^−1^, the highest feasible dose, resulted in only a 90% recovery rate (Figure 6d; Table S7, Supporting Information), underscoring its suboptimal therapeutic profile. Collectively, these results indicate that Nisin‐OCT@RBP_Sb1_, Nisin‐PA@RBP_Sb1_, and Nisin@UPSN@RBP_Sb1_ are promising candidates for treating antibiotic‐resistant *S. aureus* infections.

To further validate these findings, bacterial load measurements were conducted on the lungs and other major organs of the infected mice. As depicted in Figure 7e–i, treatment with 4 mg kg^−1^ free nisin resulted in only a slight reduction in MRSA populations. Despite the gRBP_Sb1_ absent nanomedicines, Nisin‐OCT, Nisin‐PA, and Nisin@UPSN, significantly reduced the MRSA populations in the lungs and other major organs of the infected mice, the MRSA populations of organs remained at considerably high levels. Conversely, Nisin‐OCT@RBP_Sb1_, Nisin‐PA@RBP_Sb1_, and Nisin@UPSN@RBP_Sb1_ markedly decreased MRSA populations in both the lungs and other major organs to significantly low levels. These results affirm the promising therapeutic efficacy of the bacteriophage‐mimicking nanomedicines.

## 3 Discussion

The growing threat of antibiotic resistance, coupled with a dwindling pipeline for new antibiotics, presents a serious public health challenge.^[^ ^1^, ^2^, ^3^ ^]^ This situation necessitates the development of novel therapeutics that target antibiotic‐resistant pathogens.^[^ ^52^, ^53^ ^]^ Nisin, a promising peptide antibiotic, exhibits potent bactericidal effects through a mechanism distinct from those of clinically used antibiotics.^[^ ^23^, ^24^ ^]^ However, its cationic properties contribute to hemolysis and cytotoxicity, limiting its clinical application. In this study, we developed three nanodelivery systems that emulate the precise genome delivery mechanisms used by bacteriophages. These systems significantly reduced the acute toxicity of nisin, making it suitable for intravenous administration, and markedly improved its therapeutic efficacy in an MRSA‐induced mouse pneumonia model.

Prior to our studies, bacteria‐targeting strategies for nanodelivery systems were largely based on nonspecific electrostatic interactions, ligand‐receptor interactions, and antigen‐antibody recognition.^[^ ^34^, ^54^, ^55^ ^]^ Among these, ligand‐receptor interactions and antigen‐antibody recognition demonstrated superior specificity and efficacy against bacterial pathogens.^[^ ^56^, ^57^ ^]^ Lehar et al., for instance, reported that an S. aureus‐specific antibody significantly enhanced the efficacy of rifalogue, a rifampicin derivative, against systemic MRSA infections in mice.^[^ ^56^ ^]^ Despite these promising results, large‐scale use of antibody conjugates is constrained by high production costs, as each rifalogue molecule requires a costly antibody. Additionally, while antibodies offer high specificity, they are sensitive to environmental factors such as pH, temperature, and bacterial proteases,^[^ ^58^, ^59^ ^]^ potentially complicating their use in the complex microenvironment of infection sites.

More recently, a nanodelivery system utilizing ligand‐receptor interactions and offering high antibiotic loading capacity was developed to target *S. aureus*.^[^ ^57^ ^]^ Using a phage display‐based high‐throughput screening system, researchers engineered the cyclic 9‐amino acid peptide CARG to exhibit strong affinity for *S. aureus* in a pneumonia model.^[^ ^57^ ^]^ This CARG peptide was then applied to coat biocompatible porous silicon nanoparticles (pSiNPs), which, when loaded with vancomycin, demonstrated significantly improved therapeutic efficacy over the free antibiotic in an *S. aureus*‐induced pneumonia model.^[^ ^57^ ^]^ Although the CARG‐pSiNP conjugate addressed the payload limitations, engineering similar cyclic peptides for other pathogens presents challenges due to the structural constraints of small peptides, which limit design flexibility in specificity and affinity.

In the present study, our nanodelivery systems incorporated RBPs as targeting modules, resulting in exceptional specificity and efficacy in pathogen targeting with efficient accumulation at infection sites, closely mimicking bacteriophage targeting capabilities. To further replicate the controlled genome release characteristic of bacteriophages, peptide antibiotics were integrated into the nanodelivery systems via dynamic covalent bonds. This configuration enabled infection microenvironment‐responsive drug release, achieving bacteriophage‐like precision in payload delivery. Collectively, these results confirm the successful development of bacteriophage‐mimicking nanodelivery systems.

Biosafety evaluations revealed that these nanodelivery systems effectively circumvented the acute toxicity typically associated with peptide antibiotics, making them viable for intravenous use. In comparison, free nisin at its maximum feasible dose of 32 mg kg^−1^ achieved only a 90% recovery rate, while the bacteriophage‐inspired nanodelivery systems achieved a 100% recovery rate with a significantly lower dose of 4 mg kg^−1^. This outcome underscores the successful transformation of nisin into an effective therapeutic agent against antibiotic‐resistant *S. aureus* via bacteriophage‐mimicry strategies.

The nanodelivery systems developed in this study introduce new possibilities for converting peptide antibiotics into viable intravenous therapeutics. These systems can be adapted to target a range of bacterial pathogens by incorporating specific bacteriophage RBPs, and the payload can be substituted with different peptide antibiotics to address particular bacterial threats. Overall, this study demonstrates that bacteriophage mimicry is a promising strategy for minimizing side effects and enhancing the therapeutic efficacy of peptide antibiotics, offering a viable approach to treating antibiotic‐resistant infections.

## 4 Experimental Section

Additional materials & methods are available in Supporting Information.

### Ethics Statement

All animal experiments were conducted in accordance with the Guide for the Care and Use of Laboratory Animals from the National Institutes of Health, and all procedures were approved by the Animal Research Committee of Sichuan Agricultural University (Approval No. 20240072). The use of biological materials from New Zealand white rabbits (erythrocytes isolated from the blood of healthy rabbits) was approved by the Sichuan Agricultural University Institutional Review Board.

### Bacterial Strains Used and Growth Conditions

All bacterial strains used in this study are listed in Table S3 (Supporting Information). *E. coli* TOP10 and *E. coli* BL21(DE3) were cultured on Luria–Bertani (LB) agar plates containing 1% (wt/vol) agar or in LB broth at 37 °C, with 20 µg mL^−1^ kanamycin for selection. ESKAPE pathogens were grown in LB broth at 37 °C with aeration at 220 rpm to prepare overnight cultures.

### Animals

Six‐week‐old female SPF‐grade ICR mice were purchased from Chengdu Dossy Experimental Animals Co., Ltd. The mice were housed in the Animal Center of the College of Veterinary Medicine, Sichuan Agricultural University, under standard conditions with ad libitum access to food and water. The light cycle was maintained from 7:30 am to 7:30 pm, with the temperature controlled at 22±2 °C and humidity at 40–70%. All experimental procedures involving animals were conducted in accordance with the guidelines of the Animal Care and Use Committee of Sichuan Agricultural University. Animal experiments were conducted independently using different cohorts of mice.

### Molecular Biology Techniques

Plasmids and oligonucleotide primers used in this study are listed in Tables S8 and S9, respectively. All oligonucleotide primers and inserts were purchased from Chengdu Youkangjianxing Biotechnology Co., Ltd. (Chengdu, China). The *RBP_sb1_* gene and the expression vector gene (*pRSF‐Cys‐His6‐GFP*) were amplified using 2×TransStart FastPfu PCR SuperMix (Cat. No. AS221‐02, TransGen Biotech Co., Ltd., Beijing, China). The *RBP_sb1_* gene was then inserted into *pRSF‐Cys‐His6‐GFP* using the pEASY‐Basic Seamless Cloning and Assembly Kit (Cat. No. CU201‐03, TransGen Biotech Co., Ltd., Beijing, China) to generate the gRBP_sb1_ coding plasmid *pRSF‐Cys‐His6‐GFP‐RBP_sb1_*. The sequence of the constructed plasmid was subsequently verified by sequencing at Chengdu Youkangjianxing Biotechnology Co., Ltd. (Chengdu, China).

### Expression, Purification, and Characterization of gRBP_sb1_

After verifying the correct sequence, pRSF‐Cys‐His6‐GFP‐RBP_sb1_ was transformed into *E. coli* BL21(DE3). The transformed *E. coli* was then inoculated into 20 mL of LB medium supplemented with 20 µg mL^−1^ kanamycin and incubated overnight at 37 °C with aeration at 220 rpm. The overnight culture was subsequently diluted 50‐fold into 1 L of TSB medium, also supplemented with 20 µg mL^−1^ kanamycin, and grown at 37 °C until reaching an OD_600_ of 0.6. The culture was then chilled in ice water for 10 min, and gRBP_sb1_ expression was induced by adding IPTG to a final concentration of 0.5 mm.

After induction, the culture was incubated at 18 °C for 24 h with aeration at 220 rpm. The cells were then harvested by centrifugation at 6,000 g for 15 min. The resulting cell pellets were resuspended in lysis buffer (50 mM Tris‐HCl, 2 mM EDTA, 100 mM NaCl, 0.5% Triton X‐100, pH 8.5) and sonicated for 20 min. The lysate was clarified by centrifugation at 12,000 g for 20 min, and the supernatant was filtered through a 0.45 µm membrane. The clarified lysate was applied to a Ni‐NTA agarose column (Beijing Solarbio & Technology Co., Ltd., Beijing, China) equilibrated with buffer (50 mM NaH2PO_4_, 500 mM NaCl, 10 mM imidazole, pH 8.0). After discarding the flow‐through, the column was washed with 12 column volumes (CV) of wash buffer (50 mM NaH2PO_4_, 500 mM NaCl, 20 mM imidazole, pH 8.0), and gRBP_sb1_ was eluted with 6 CV of elution buffer (50 mM NaH2PO_4_, 500 mM NaCl, 500 mM imidazole, pH 8.0). After that, the gRBP_sb1_ protein was further purified using a GE prepacked gel filtration column (HiPrep™ 16/60 Sephacryl® S‐200 HR, GE17‐1166‐01).

Finally, the purified gRBP_sb1_ was analyzed by 8% SDS‐PAGE and visualized with Coomassie Blue staining. Moreover, Western blot analysis was performed on the purified gRBP_sb1_ using a 1:20,000 dilution of His‐Tag (6^*^His) monoclonal antibody and a 1:5,000 dilution of HRP‐conjugated AffiniPure Goat Anti‐Mouse IgG(H+L).

### Preparation of Bacteriophage‐Mimicking Nanomedicines—General Procedure for Preparing Dynamic Covalent Amphiphile Assemblies

The dynamic covalent amphiphile assemblies were prepared following the protocol outlined in our previous study.^[^ ^38^ ^]^ As an example, Nisin‐OCT assemblies were prepared by adding 400 µL of nisin stock solution (10 mg mL^−1^) and 60 µL of (E)‐2‐Octenal (OCT) stock solution dropwise into 1540 µL of pH 8.5 phosphate buffer solution (10 mM) while stirring at 4 °C. After stirring for 30 min, the Nisin‐OCT assemblies were isolated by centrifugation at 12,000 g for 30 min. Similar procedures were used for the preparation of other assemblies.

### Coupling of gRBP_Sb1_ to Nisin‐OCT Assemblies

Nisin‐OCT assemblies were resuspended in 2 mL of 0.1 m phosphate buffer (pH 7.4). To this suspension, 0.05 mg of MAL‐PEG‐NHS was added and the mixture was incubated at room temperature with stirring for 1 h to generate MAL‐PEG conjugated Nisin‐OCT assemblies. Following the removal of excess reagent by centrifugation at 12,000 g for 30 min, the maleimide‐activated Nisin‐OCT assemblies were resuspended in 2 mL of 0.1 m phosphate buffer (pH 7.4). The suspension was then mixed with 60 µL of gRBP_Sb1_ (8 mg mL^−1^) and stirred for 2 h at room temperature to conjugate gRBP_Sb1_ via the free cysteine residue at the N‐terminus of the protein, resulting in the formation of Nisin‐OCT@RBP_Sb1_. After centrifugation at 12,000 g for 30 min to remove excess proteins, the Nisin‐OCT@RBP_Sb1_ was washed three times with PBS. Finally, the Nisin‐OCT@RBP_Sb1_ was lyophilized and stored at ‐20 °C until use. For resuspension before administration, sterilized sodium chloride solution or phosphate‐buffered saline is recommended.

### General Procedure for Preparing Dynamic Covalent Nano‐Networks

Dynamic covalent nano‐networks were prepared using the method described in the previous study.^[^ ^39^ ^]^ As an example, Nisin‐PA nano‐networks were synthesized as follows: PVP (18 mg) was dissolved in 7.60 mL of carbonate buffer (pH 8.5, 50 mM). Stock solutions of 4‐ABBA in DMSO (176 µL, 10 mg mL^−1^) and nisin in ultrapure water (900 µL, 10 mg mL^−1^) were then added to the buffer at room temperature with magnetic stirring (2500 rpm). Procyanidin in DMSO (320 µL, 10 mg mL^−1^) was added dropwise at a rate of 1 drop per 10 s, resulting in a final NH_2_/C═O/catechol molar ratio of 1:1:1 and a final nisin concentration of 1 mg mL^−1^. The suspension became turbid upon the addition of polyphenols and was stirred at room temperature for 1 h to form Nisin‐PA nano‐networks. The freshly prepared nanoparticles were separated by centrifugation at 12,000 g for 30 min. Similar procedures were used to prepare other nano networks.

### Coupling of RBP_Sb1_ to Nisin‐PA Nano‐Networks

The Nisin‐PA nano‐networks were resuspended in 2 mL of 0.1 m phosphate buffer (pH 7.4). Subsequently, 0.1 mg of MAL‐PEG‐NHS was added to the suspension and incubated at room temperature for 1 h with stirring to generate MAL‐PEG‐conjugated Nisin‐PA nano‐networks. After centrifugation at 12,000 g for 30 min to remove excess reagent, the maleimide‐activated Nisin‐PA nano‐networks were mixed with 200 µL of RBP_Sb1_ (8 mg mL^−1^) and stirred for 2 h at room temperature to conjugate RBP_Sb1_ via the free cysteine residue at the N‐terminus of the protein, generating Nisin‐PA@RBP_Sb1_. Excess proteins were removed by centrifugation at 12,000 g for 30 min, and the engineered Nisin‐PA@RBP_Sb1_ was washed with PBS three times. The Nisin‐PA@RBP_Sb1_ was then lyophilized and stored at −20 °C until use. A sterilized sodium chloride solution or phosphate‐buffered saline is recommended for resuspension of the nanomedicine before administration.

### General Procedure for Preparing Nisin@UPSN and Nisin@UPSN@RBP_Sb1_

Aminated urchin‐like porous silica nanoparticles (UPSNs‐NH₂) were synthesized using the method described in previous studies.^[^ ^31^, ^46^ ^]^ UPSNs‐NH₂ were resuspended in 1 mL of 0.1 m phosphate buffer (pH 7.4) to a final concentration of 5 mg mL^−1^. Subsequently, 0.15 mg of MAL‐PEG‐NHS and 3 mg of CHO‐PEG‐NHS (molar ratio 1:100) were added to the suspension and incubated at room temperature for 1 h with stirring to generate MAL‐PEG‐ and CHO‐PEG‐conjugated UPSNs. After removing excess reagents by centrifugation at 8,000 g for 10 min, the maleimide‐ and aldehyde‐activated UPSNs were resuspended in 1 mL of 0.1 m phosphate buffer (pH 7.4). The activated UPSNs were then mixed with an aqueous solution of nisin (1 mL, 10 mg mL^−1^) and incubated at room temperature for 1 h with magnetic stirring at 1,000 rpm. Following this, excess reagents were removed by centrifugation at 8,000 g for 10 min, yielding Nisin@UPSN. The Nisin@UPSN was subsequently resuspended in 2 mL of 0.1 M phosphate buffer (pH 7.4) and mixed with 200 µL of gRBP_Sb1_ (8 mg mL^−1^). The mixture was stirred for 2 h at room temperature to conjugate gRBP_Sb1_ via the free cysteine residue at the N‐terminus of the protein, forming Nisin@UPSN@RBP_Sb1_. Excess proteins were removed by centrifugation at 8,000 g for 10 min, and the engineered Nisin@UPSN@RBP_Sb1_ was washed with PBS three times. The Nisin@UPSN@RBP_Sb1_ was then lyophilized and stored at −20 °C until use. A sterilized sodium chloride solution or phosphate‐buffered saline is recommended for resuspension of the nanomedicine before administration.

### Characterization of Bacteriophage‐Mimicking Nanomedicines—Dynamic Light Scattering and Zeta Potential Measurements

Hydrodynamic diameters and zeta potentials of bacteriophage‐mimicking nanomedicines, as well as nanomedicines without gRBP_sb1_, were measured at a concentration of 1 mg mL^−1^ in ultrapure water using a Malvern Zetasizer Nano ZSE (Malvern, UK).

### Transmission Electron Microscopy (TEM)

The morphologies and elemental distributions of the synthesized nanomedicines were analyzed using a Talos F200E TEM (Thermo Fisher Scientific, USA) operating at an accelerating voltage of 200 kV.

### Fourier Transform Infrared Spectroscopy (FT‐IR)

FT‐IR measurements were conducted on lyophilized samples using a Spectrum Two FT‐IR Spectrometer with a LiTa detector (PerkinElmer, UK).

### Confocal Laser Scanning Microscopy (CLSM)

CLSM was employed to confirm the correct conjugation of gRBP_sb1_ on the bacteriophage‐mimicking nanomedicines. Briefly, the loading module of the nanomedicines was first labeled with DyLight 633, a red fluorescent dye. Subsequently, gRBP_sb1_ was conjugated to the nanomedicines. The DyLight 633‐labeled nanomedicines were then visualized using a STELLARIS STED/EM CPD300 confocal microscope (Leica, Germany).

### *S. aureus* Specific Targeting Capacity Analysis

The binding specificity of gRBP_sb1_ to *S. aureus* was assessed using fluorescence microscopy. Briefly, the cell density of ESKAPE pathogens was adjusted to an OD_600_ of 0.2 in PBS, followed by the addition of gRBP_sb1_ to a final concentration of 20 µg mL^−1^. After a 30‐min incubation at 37 °C, the cell suspensions were washed three times with PBS, loaded onto 1.5% agarose pads, and examined using a Nikon 80i microscope (Japan).

Additionally, MRSA (ATCC 43300) and *E. coli* (ATCC 25922) cells were stained with DAPI at a final concentration of 1 mg mL^−1^ and incubated at 37 °C for 2 h. The cells were then treated with either gRBP_sb1_ (20 µg mL^−1^) or DyLight 633‐labeled bacteriophage‐mimicking nanomedicines (200 µg mL^−1^) at 37 °C for 30 min. After three PBS washes, the samples were loaded onto 1.5% agarose pads and analyzed using a STELLARIS STED/EM CPD300 confocal microscope (Leica, Germany).

### gRBP_sb1_‐Mediated MRSA Infection Site Targeting

SPF‐grade ICR female mice (6 weeks old, 20 ± 2 g, n = 3 per group) were intratracheally inoculated with MRSA (ATCC 43300) at a dose of 6 × 10⁹ c.f.u. per mouse to establish the MRSA‐induced lung infection model. At 24 h post‐infection, the mice received either gRBP_sb1_ (0.5 mg per mouse) or bacteriophage‐mimicking nanomedicines (4 mg per mouse) via intravenous injection. Mice without MRSA infection served as controls and were treated with gRBP_sb1_ (0.5 mg per mouse), bacteriophage‐mimicking nanomedicines (4 mg per mouse), or 0.1 M PBS. After 30 min of circulation, the mice were sacrificed, and their lungs were harvested. Fluorescence images of the lungs were captured using a FUSION FX7 EDGE Imaging System.

### Biosafety Assessment—Hemolytic Activity Assay

Hemolytic activity was assessed using the method described in previous studies.^[^ ^19^, ^31^ ^]^ Briefly, erythrocytes were freshly isolated from the blood of healthy rabbits and washed three times with 0.1 M PBS. Nisin, Nisin‐OCT, Nisin‐OCT@RBP_Sb1_, Nisin‐PA, Nisin‐PA@RBP_Sb1_, Nisin@UPSN, or Nisin@UPSN@RBP_Sb1_ were then added to the erythrocytes at final concentrations of 1024, 512, 128, 64, 32, 16, 8, and 0 µg mL^−1^ in PBS containing 2% (v/v) erythrocytes. The cells were incubated at 37 °C for 1 h, then centrifuged at 3000 g for 15 min. The supernatant was transferred to a 96‐well plate, and absorbance was measured at 570 nm using a Thermo Scientific Varioskan Flash multimode microplate reader. Hemolysis was quantified as the percentage of absorbance relative to the positive control, which was treated with 10% Triton X‐100. Representative results from three independent replicates are shown.

### Mammalian Cytotoxicity

The cytotoxicity of nisin, Nisin‐OCT, Nisin‐OCT@RBP_Sb1_, Nisin‐PA, Nisin‐PA@RBP_Sb1_, Nisin@UPSN, and Nisin@UPSN@RBP_Sb1_ was evaluated using the CCK‐8 Cell Proliferation and Cytotoxicity Assay Kit (Cat. No. CA1210, Solarbio, China). The assay was conducted on two cell lines: the hepatoblastoma cell line (Hep G2) (ATCC HB‐8065) and human embryonic kidney 293T cells (HEK‐293T, ATCC CRL‐3216). HepG2 and HEK‐293T cells were cultured in Dulbecco's Modified Eagle's Medium (DMEM) supplemented with 10% fetal bovine serum (FBS) and seeded into 96‐well plates. After 24 h of incubation at 37 °C with 5% CO₂, the medium was replaced with fresh DMEM containing 2% FBS and different concentrations of nisin or nanoparticles (100 µL per well). Following an additional 24‐h incubation, CCK‐8 reagent was added according to the manufacturer's instructions, and the plates were incubated at 37 °C for 2 h with 5% CO₂. Absorbance was measured at 450 nm using a Thermo Scientific Varioskan Flash multimode microplate reader.

### In Vivo Toxicity Assessments

One hundred seventy healthy SPF‐grade ICR mice (female, 6 weeks old, 20 ± 2 g) were randomly divided into seventeen groups (n = 10 per group) and treated with free nisin, Nisin‐OCT@RBP_Sb1_, Nisin‐PA@RBP_Sb1_, or Nisin@UPSN@RBP_Sb1_ at doses of 32, 64, 128, or 256 mg kg^−1^, respectively. Mice treated with sterile saline served as the untreated control. Mortality rates for each treatment group were monitored over a period of 7 days.

In a separate experiment, forty additional healthy SPF‐grade ICR mice (female, 6 weeks old, 20 ± 2 g) were randomly assigned to four groups (n = 10 per group) and treated with Nisin‐OCT@RBPSb1 (40 mg kg^−1^), Nisin‐PA@RBPSb1 (40 mg kg^−1^), Nisin@UPSN@RBPSb1 (40 mg kg^−1^), or sterile saline. Body weights of the mice were recorded daily for 7 days. Following this period, the mice were euthanized, and serum and whole blood were collected for comprehensive metabolic panel and blood cell count analyses. Additionally, the hearts, livers, spleens, lungs, and kidneys were harvested, fixed in 4% paraformaldehyde, sectioned into 5 µm slices, and stained with hematoxylin and eosin (H&E) for histological examination.

### Therapeutic Efficacy Assessment

SPF‐grade ICR female mice (6 weeks old, 20±2 g, n = 10 per group) were intratracheally inoculated with MRSA (ATCC 43300) at a dose of 7 × 10⁹ c.f.u. per mouse, resulting in 90% mortality within 12–72 h post‐infection. At 12 h post‐infection, the mice were administered Nisin‐OCT@RBP_Sb1_ (8, and 4 mg kg^−1^, or 2 mg kg^−1^), Nisin‐OCT (4 mg kg^−1^), Nisin‐PA@RBP_Sb1_ (8, 4 and mg kg^−1^, or 2 mg kg^−1^), Nisin‐PA (4 mg kg^−1^), Nisin@UPSN@RBP_Sb1_ (8, and 4 mg kg^−1^, or 2 mg kg^−1^), Nisin@UPSN (4 mg kg^−1^), free nisin (4 mg kg^−1^), or 0.1 M PBS via intravenous injection. Mice without MRSA infection served as non‐infection controls. Survival rates across the groups were monitored for 14 days.

In addition, another cohort of SPF‐grade ICR female mice (6 weeks old, 20±2 g, n = 6 per group) were intratracheally infected with MRSA (ATCC 43300) at a dose of 7 × 10⁹ c.f.u. per mouse. At 12 h post‐infection, the mice received Nisin‐OCT@RBP_Sb1_ (4 mg kg^−1^), Nisin‐OCT (4 mg kg^−1^), Nisin‐PA@RBP_Sb1_ (4 mg kg^−1^), Nisin‐PA (4 mg kg^−1^), Nisin@UPSN@RBP_Sb1_ (4 mg kg^−1^), Nisin@UPSN (4 mg kg^−1^), free nisin (4 mg kg^−1^), or 0.1 M PBS via intravenous injection. Mice without MRSA infection were used as non‐infection controls. At 24 h post‐infection, organs including the heart, liver, spleen, lungs, and kidneys were collected to measure bacterial loads.

### Statistical Analysis

All the statistical analyses were performed using GraphPad Prism 8 software (GraphPad Software). Data were expressed as means ± standard deviation of experiments with at least three independent assays. The statistical significance of the data was assessed using one‐way ANOVA followed by Tukey's multiple comparisons test with GraphPad Prism 8.0. Survival was analyzed using the Log‐rank (Mantel‐Cox) test with GraphPad Prism 8.0. ns, no significance; ^*^ *p* < 0.05; ^**^ *p* < 0.01; ^***^ *p* < 0.001; ^****^ *p* < 0.0001.

## Conflict of Interest

The authors declare no conflict of interest.

## Author Contributions

H.W., X. Zhong, S.Y., and J.D. contributed equally to this work. H.W. and X. Zhao conceived the project and strategies. X. Zhao, Y.L., and Y.L. supervised the work and corrected the manuscript. H.W., X. Zhong, S.Y., and J.D. designed and carried out the experiments, analyzed data, and wrote the manuscript. X.S., and Z.Y. did experimental work on animal studies. X. Zhao wrote the manuscript and analyzed the data. All authors contributed to and commented on the manuscript text and approved its final version.

## Supporting information



Supporting Information

## Data Availability

The data that support the findings of this study are available from the corresponding author upon reasonable request.
