# Supplementary material for: Enhancing the Therapeutic Potential of Peptide Antibiotics Using Bacteriophage Mimicry Strategies
Source: Adv Sci (Weinh). 2024 Nov 25;12(3):2411753. doi: 10.1002/advs.202411753 (PMC11744576; doi:10.1002/advs.202411753)
Supplement: Supplementary file 1 — Supporting Information [file ADVS-12-2411753-s001.docx]

**Supporting Information**

**Enhancing the Therapeutic Potential of Peptide Antibiotics Using Bacteriophage Mimicry Strategies**

Hongping Wan^1,2,5,^*, Xinyi Zhong^1,2,5^, Shinong Yang^1,2,5^, Jiarong Deng^1,2,5^, Xu Song^1^, Yong Liu^3,^*, Yuanfeng Li^4,^*, Zhongqiong Yin^1^, Xinghong Zhao^1,2,^*

^1^ Center for Sustainable Antimicrobials, Department of Pharmacy, Sichuan Agricultural University, Chengdu, 611130, China.

^2^ Center for Infectious Diseases Control (CIDC), Sichuan Agricultural University, Chengdu, 611130, China.

^3^ State Key Laboratory of Medicinal Chemical Biology, Nankai University, Tianjin, 300071, China.

^4^ Translational Medicine Laboratory, The First Affiliated Hospital of Wenzhou Medical University, Wenzhou, Zhejiang, 325035, China.

^5^ These authors contributed equally to this work.

* Correspondence: hpwan@sicau.edu.cn (H. Wan); y.liu@nankai.edu.cn (Y. Liu); yuanfengli@wmu.edu.cn (Y. Li); xinghong.zhao@sicau.edu.cn (X. Zhao)

**Supplementary Materials & Methods**

Reagents

2×TransStart FastPfu PCR SuperMix (Cat No. AS221-02), pEASY-Basic Seamless Cloning and Assembly Kit (Cat No. CU201-03), EasyPure Quick Gel Extraction Kit (Cat No. EG101-01), and EasyPure HiPure Plasmid MiniPrep Kit (Cat No. EM111-01) were purchased from TransGen Biotech Co., Ltd. (Beijing, China); Luria–Bertani broth (LB, Cat No. HB0128) and Tryptic Soy Broth (TSB, Cat No. HB4114) were purchased from Qingdao Hope Bio-Technology CO., Ltd. (Qingdao, China); 4',6-diamidino-2-phenylindole (DAPI, Cat No. BS097-10mg) and D8000 DNA Marker (100-8000bp) (Cat No. BL1332A) were purchased from Labgic Bioechnology Co., Ltd. (Beijing, China); Isopropyl-β-D-thiogalactopyranoside (IPTG) (Cat No. I8070), Ni-NTA agarose HP (Cat No. S9320), ColorMixed Protein Marker (11-180KDa) (Cat No. PR1910); CCK-8 Cell Proliferation and Cytotoxicity Assay Kit (Cat No. CA1210) and ECL Western Blotting Substrate (Cat No. PE0010) were purchased from Beijing Solarbio & Technology Co., Ltd. (Beijing, China); His-Tag (6*His) Monoclonal antibody (Cat No. 66005-1-lg) and HRP-conjugated Affinipure Goat Anti-Mouse IgG(H+L) (Cat No. SA00001-1) were purchased from Proteintech Group, Inc (Rosemont, USA); nisin (Cat No. 0302) was purchased from Handary (Brussels, Belgium); cetyltrimethylammonium bromide (CTAB, Cat No. 57-09-0) was purchased from Shanghai Macklin Biochemical Co., Ltd. (Shanghai China); tetraethyl orthosilicate (TEOS**,** Cat No. 78-10-4) was purchased from Sigma-Aldrich (St. Louis, USA); Maleimide-polyethylene glycol-N-hydroxylsuccinimide (MW=5000Da, MAL-PEG-NHS, Cat No. R-1083-5K) and Aldehyde-polyethylene glycol-N-hydroxylsuccinimide (MW=1000Da, CHO-PEG-NHS, Cat No. R-1050-1k) were purchased from Xi’an ruixi Biological Technology Co., Ltd. (Xi’an, China); N-hydroxysuccinimide containing red fluorescent dye DyLight 633 was purchased form ThermoFisher scientific (Cat No. 46414, St. Louis, USA); trans-2-Hexen-1-al （HEX, Cat No. H107633-25ml）, trans, trans-2,4-Heptandienal (2,4-HEP, Cat No. D107642-5ml), trans-2-Heptenal (HEP, Cat No. T161484-5ml), trans-2-Octenal (OCT, Cat No. O105936-5ml), trans-2-Nonenal (NON, Cat No. T162631-5ml), 2,4-decadienal (2,4-DEC, Cat No. D107642-5ml), trans-2-Decenal (DEC, Cat No. D117855-5ml), Citral (CIT, Cat No. C104134-25ml), trans-2-Dodecenal (DOD, Cat No. T162346-5ml), Tannic acid (TA, Cat No. T292288-25g), Proanthocyanidins (PA, Cat No. P413229-1g), (-)-Epigallocatechin gallate (EGCG, Cat No. E107404-100mg), Rosmarinic acid (RA, Cat No. R109805-250mg), Nordihydroguaiaretic Acid (NA, Cat No. 133726-250mg), Ellagic acid (EA, Cat No. E102710-1g), 4-Acetylbenzeneboronic Acid (4-ABBA, Cat No. A100554-1g), (3-aminopropyl)triethoxysilane (APTES, Cat No. A107147-100ml), and Polyvinylpyrrolidone (PVP, MW=58kDa, Cat No. P110607-100g) were purchased from Shanghai Aladdin Biochemical Techology Co., Ltd. (Shanghai China); Tribromoethanol (Cat No. M2940) was purchased from Nanjing AIBI Bio-Technology Co., Ltd. (Nanjing, China).

Characterization of Bacteriophage-mimicking Nanomedicines

**Drug loading capacity and in *vitro* release profile.** The total amount of unencapsulated nisin was determined using high-performance liquid chromatography (HPLC). The total amount of encapsulated nisin was calculated using the following equation: Total content of encapsulated nisin = Total content of nisin - Total content of unencapsulated nisin. The total content of nanomedicines was measured using an electronic balance after lyophilization. The loading capacity (LC) was calculated using the formula: LC% = (Total content of encapsulated nisin / total content of nanomedicines) × 100%. The encapsulation efficiency (EE) was determined using the formula: EE% = (Total content of encapsulated nisin / Total content of nisin) × 100%.

Nisin release profiles from different nanomedicines were monitored over 72 hours. Briefly, 5 mg of each sample (Nisin-OCT, Nisin-OCT@RBP_Sb1_, Nisin-PA, Nisin-PA@RBP_Sb1_, Nisin@UPSN, or Nisin@UPSN@RBP_Sb1_) was dispersed in 5 mL of PBS (pH 5.0 or pH 7.4) at 37 °C with shaking at 60 rpm. At specified time points, 2 mL of the supernatant was removed after centrifugation and replaced with 2 mL of fresh PBS at the corresponding pH. Nisin release was analyzed by HPLC, and the drug release profile was plotted accordingly.

Nisin concentration in solution samples was measured using an Agilent 1260 Infinity HPLC system equipped with a Phenomenex Aeris™ C18 column (250 × 4.6 mm, 3.6 μm particle size, 100 Å pore size). The mobile phase consisted of acetonitrile with a gradient of 28-40% aqueous acetonitrile (aq. MeCN) over 20 minutes at a flow rate of 1 mL/min. The gradient was as follows: 0-3 minutes, 10%-28% aq. MeCN; 3.01-13 minutes, 28%-40% aq. MeCN; 13.01-14 minutes, 40%-95% aq. MeCN; 14.01-17 minutes, 95% aq. MeCN; 17-17.01 minutes, 95%-10% aq. MeCN; 17.01-20 minutes, 10%-10% aq. MeCN. Solvent A was Milli-Q water with 0.1% TFA, and Solvent B was acetonitrile with 0.1% TFA. The UV detector was set to 214 nm to monitor changes in nisin concentration, and the column temperature was maintained at 30 °C during the analysis.

**Quantification of gRBP_Sb1_ on the bacteriophage-mimicking nanomedicines.** The quantity of gRBP_Sb1_ on bacteriophage-mimicking nanomedicines was determined using a fluorescence-based method with a standard curve generated from known concentrations of gRBP_Sb1_. To create this curve, gRBP_Sb1_ was diluted in 200 µL of PBS at final concentrations of 256, 128, 64, 32, and 16 µg/mL. Bacteriophage-mimicking nanomedicines samples were similarly prepared, resuspended in 200 µL of PBS at appropriate final concentrations, with gRBP_Sb1_-free nanomedicine samples serving as blank controls. Fluorescence measurements were obtained using a Thermo Scientific Varioskan Flash multimode microplate reader, with an excitation wavelength of 395 nm and an emission wavelength of 509 nm.

**Supplementary Figures**


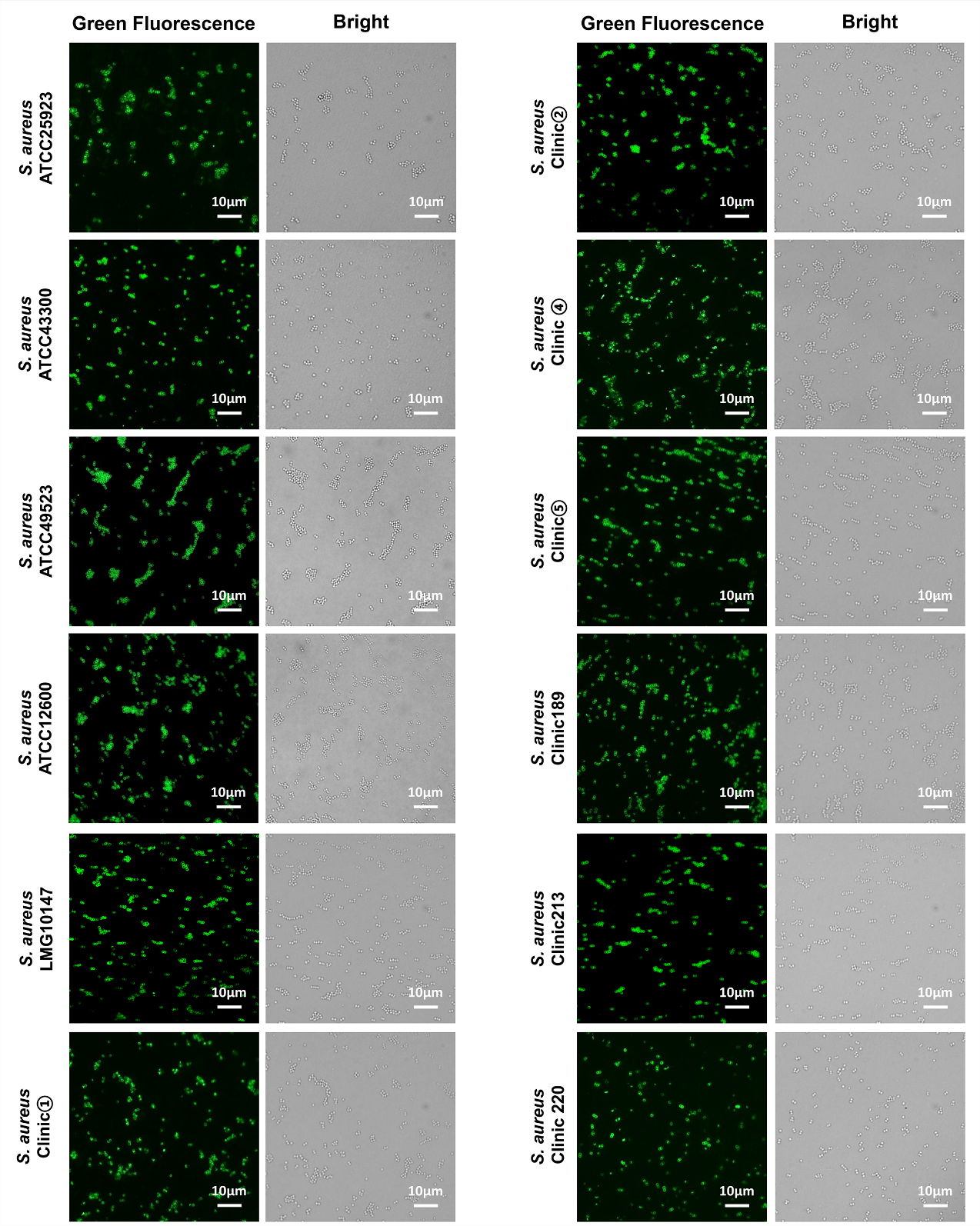


**Figure S1**: Fluorescence microscopy images of *S. aureus* cells after treatment of gRBP_sb1_ at 37 °C for 30 min. The observed green cells demonstrate that gRBP_sb1_ was bonded to all twelve tested *S. aureus* strains. (Scale bars represent 10 μm). Three times the experiment was repeated with similar results.


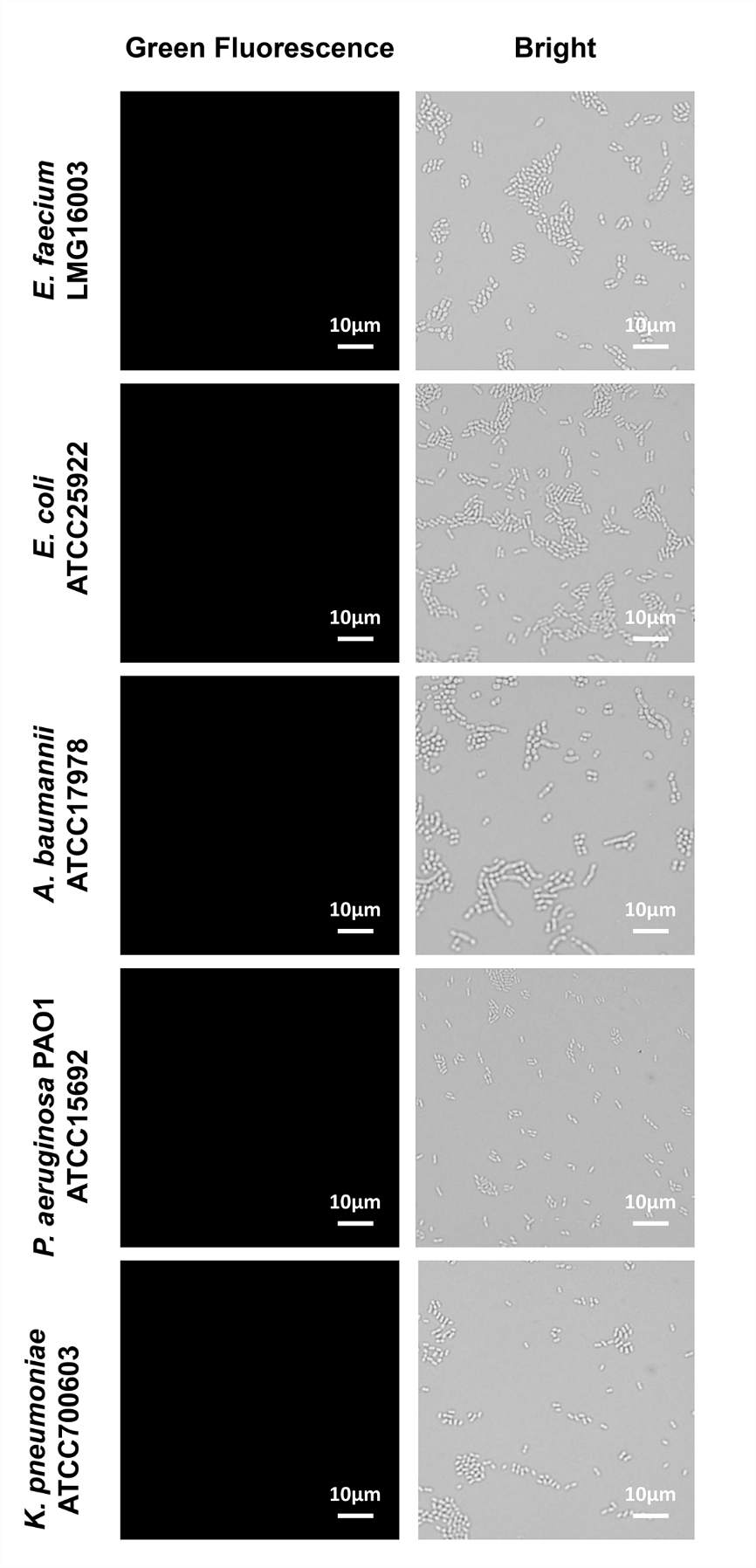


**Figure S2**: Fluorescence microscopy images of *E. faecium*, *K. pneumoniae*, *A. baumannii*, *E. coli*, and *P. aeruginosa* PAO1 after treatment of gRBP_sb1_ at 37 °C for 30 min. No green cells were observed, demonstrating gRBP_sb1_ was specifically bonded to *S. aureus* strains. (Scale bars represent 10 μm). Three times the experiment was repeated with similar results.


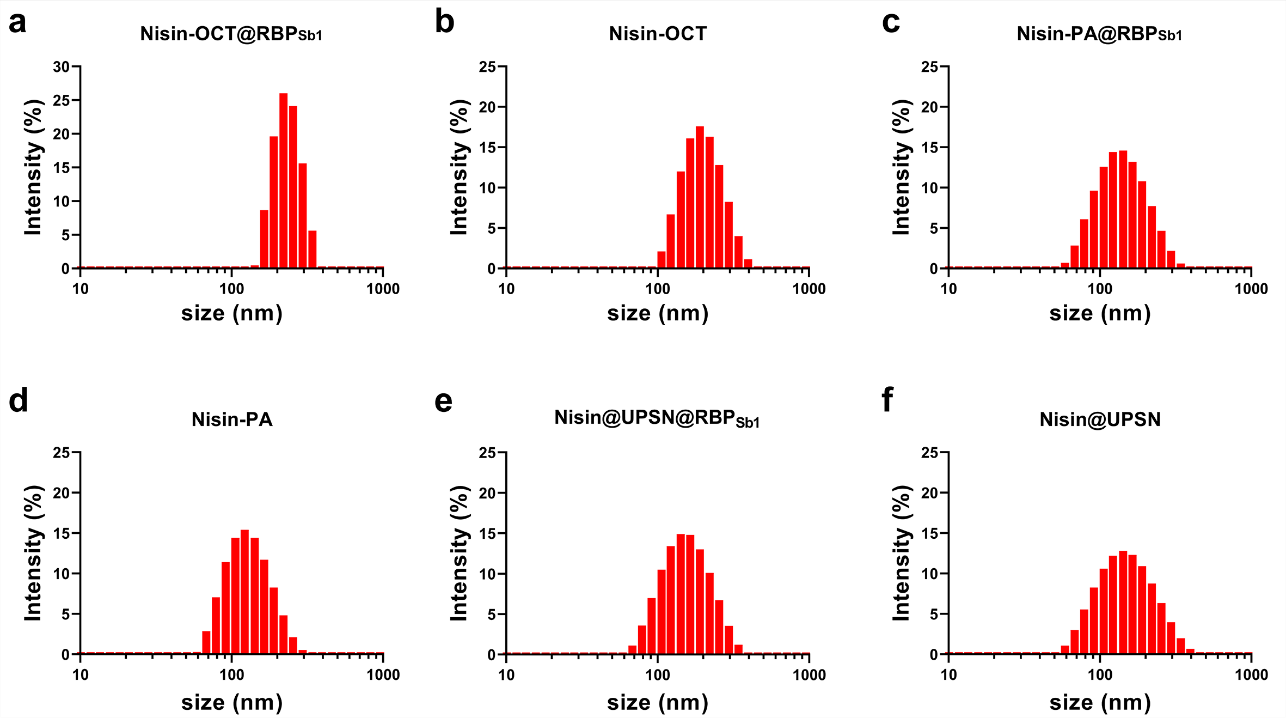


**Figure S3**: Hydrodynamic size distribution of the nanomedicines measured by dynamic light scattering. **a**, Nisin-OCT@RBP_Sb1_; **b**, Nisin-OCT; **c**, Nisin-PA@RBP_Sb1_; **d**, Nisin-PA; **e**, Nisin@UPSN@RBP_Sb1_; **f**, Nisin@UPSN.


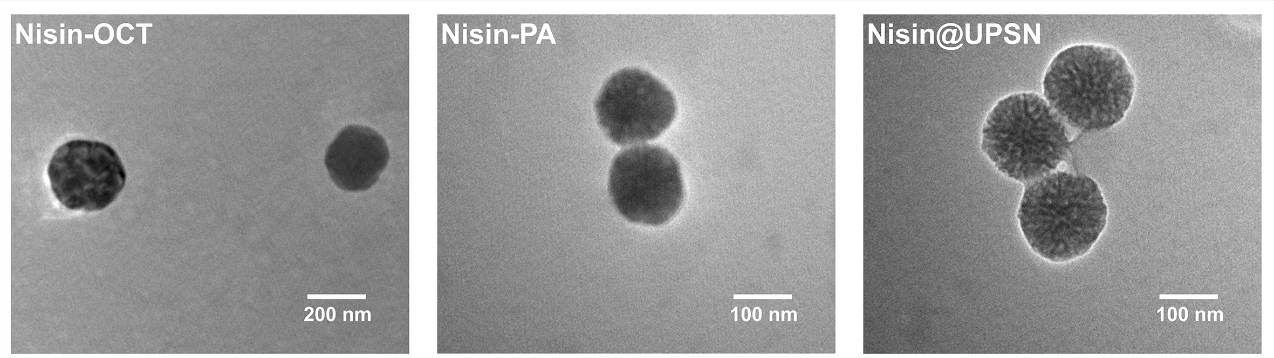


**Figure S4**: Transmission electron microscope images of Nisin-OCT, Nisin-PA, and Nisin@UPSN.


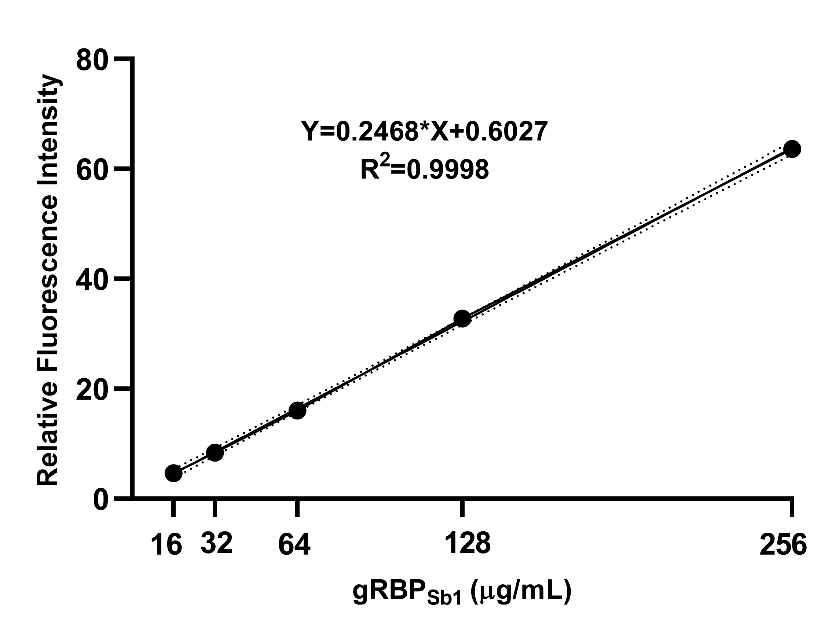


**Figure S5**: Standard fluorescence intensity curve of gRBP_sb1_.


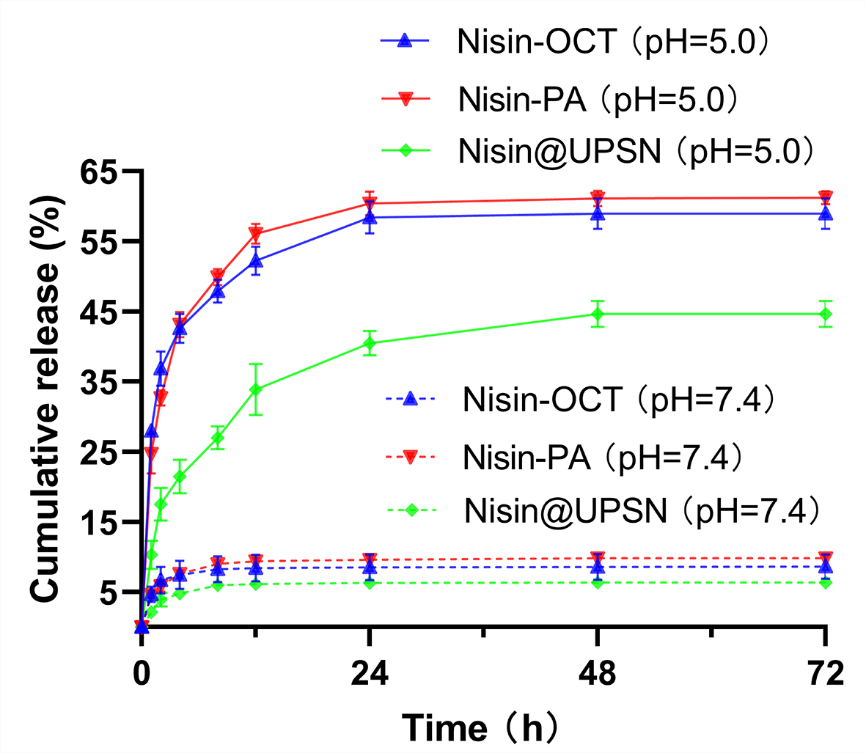


**Figure S6**: Release profiles of nisin payload from the nanomedicines in PBS (pH=5.0 or pH=7.4) at 37 °C. Data are presented as mean ± standard deviation (n = 3).


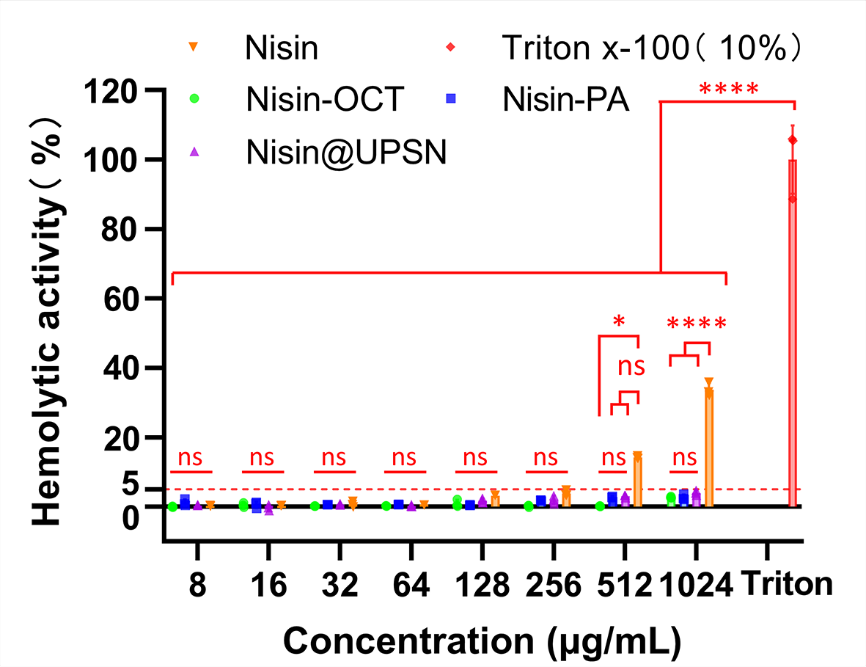


**Figure S7**: Hemolytic activity of free nisin, Nisin-OCT, Nisin-PA, and Nisin@UPSN. Data are presented as mean ± standard deviation (n=3 biological replicates). The statistical significance of the data was assessed using one-way ANOVA followed by Tukey's multiple comparisons test. ns, no significance; *p < 0.05; ****p < 0.0001.


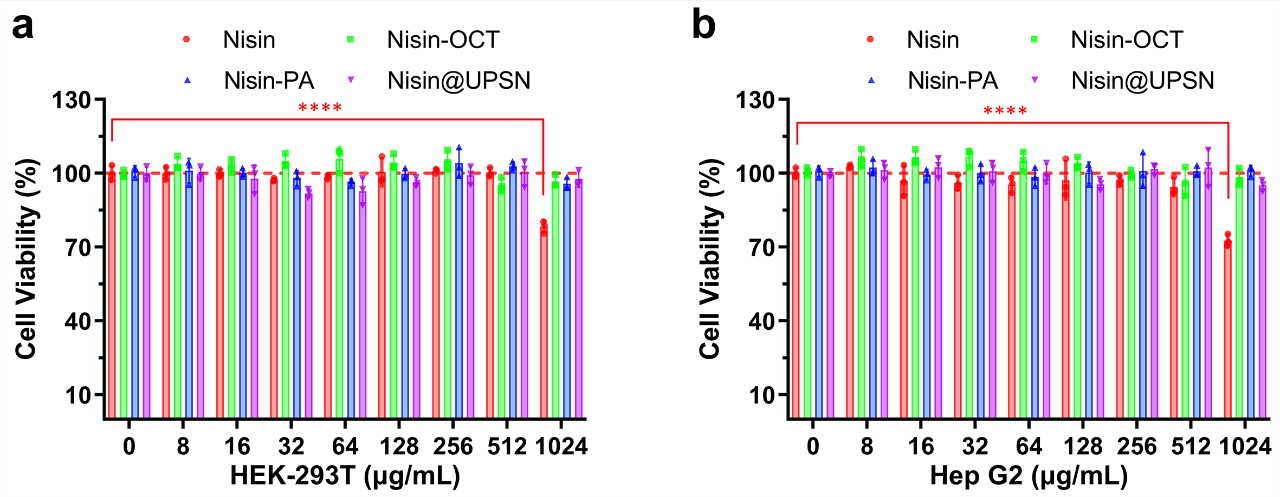


**Figure S8**: Cytotoxicity of free nisin, Nisin-OCT, Nisin-PA, and Nisin@UPSN. Viability of HEK-293T (**a**) and Hep G2 (**b**) after treatment with nisin, Nisin-OCT, Nisin-PA, or Nisin@UPSN at concentrations ranging from 16 to 1024 μg/mL for 24 h. Data are presented as mean ± standard deviation (n=3 biological replicates). The statistical significance of the data was assessed using one-way ANOVA followed by Tukey's multiple comparisons test. ns, no significance; ****p < 0.001 vs. untreated cells.


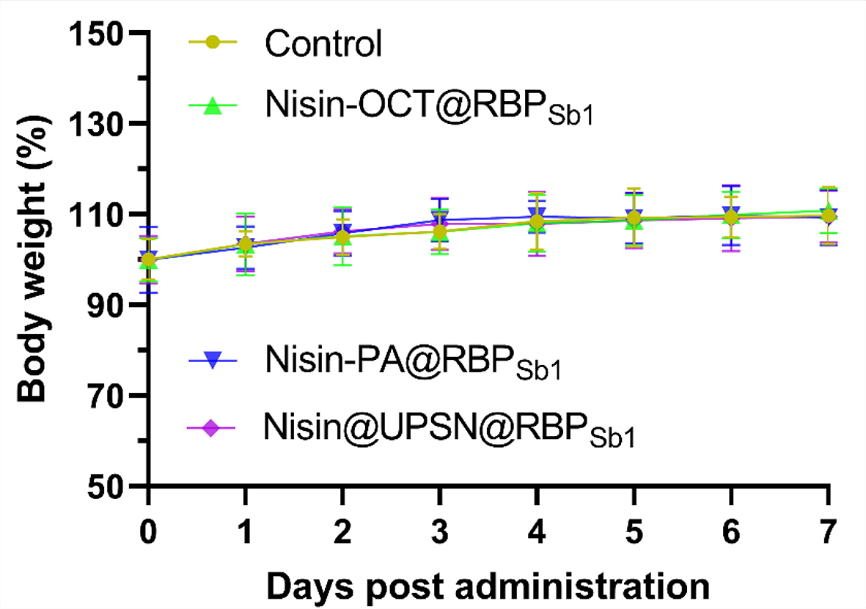


**Figure S9**: Body weight changes were recorded after treatment with Nisin-OCT@RBP_Sb1_, Nisin-PA@RBP_Sb1_, Nisin@UPSN@RBP_Sb1_, or PBS over 7 d. Data are presented as mean ± standard deviation (n=10 biological replicates).


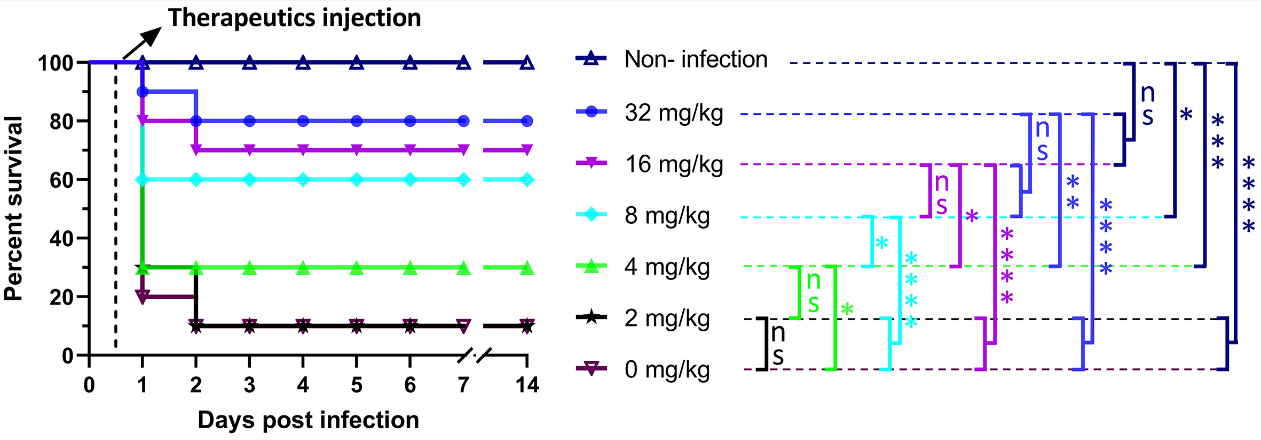


**Figure S10**: Survival rate (n= 10 biological replicates) of mice after intratracheal inoculation of MRSA at a dose of 7×10^9^ c.f.u. per mouse. Groups of mice received free nisin at a dose range of 2 to 32 mg/kg intravenously 12 h post the bacterial inoculation. Survival was analyzed by the Log-rank (Mantel-Cox) test. ns, no significance; *p < 0.05; **p < 0.01; ***p < 0.001; ****p < 0.0001.

**Supplementary Tables**

**Table S1**: Gene sequence of RBP_Sb1_.

| Gene | Sequence |
| --- | --- |
| RBP_Sb1_ | ATGGCGCTGAACTTTACCACCATTACCGAAAACAACGTGATTCGCGATCTGACCACCCAGGTGAACAACATTGGCGAAGAACTGACCAAAGAACGCAACATTTTTGATATTACCGATGATCTGGTGTATAACTTTAACAAAAGCCAGAAAATTAAACTGACCGATGATAAAGGCCTGACCAAAAGCTATGGCAACATTACCGCGCTGCGCGATATTAAAGAACCGGGCTATTATTATATTGGCGCGCGCACCCTGGCGACCCTGCTGGATCGCCCGGATATGGAAAGCCTGGATGTGGTGCTGCATGTGGTGCCGCTGGATACCAGCAGCAAAGTGGTGCAGCATCTGTATACCCTGAGCACCAACAACAACCAGATTAAAATGCTGTATCGCTTTGTGAGCGGCAACAGCAGCAGCGAATGGCAGTTTATTCAGGGCCTGCCGAGCAACAAAAACGCGGTGATTAGCGGCACCAACATTCTGGATATTGCGAGCCCGGGCGTGTATTTTGTGATGGGCATGACCGGCGGCATGCCGAGCGGCGTGAGCAGCGGCTTTCTGGATCTGAGCGTGGATGCGAACGATAACCGCCTGGCGCGCCTGACCGATGCGGAAACCGGCAAAGAATATACCAGCATTAAAAAACCGACCGGCACCTATACCGCGTGGAAAAAAGAATTTGAACCGAAAGATATGGAAAAATATCTGCTGAGCAGCATTCGCGATGATGGCAGCGCGAGCTTTCCGCTGCTGGTGTATACCAGCGATAGCAAAACCTTTCAGCAGGCGATTATTGATCATATTGATCGCACCGGCCAGACCACCTTTACCTTTTATGTGCAGGGCGGCGTGAGCGGCAGCCCGATGAGCAACAGCTGCCGCGGCCTGTTTATGAGCGATACCCCGAACACCAGCAGCCTGCATGGCGTGTATAACGCGATTGGCACCGATGGCCGCAACGTGACCGGCAGCGTGGTGGGCAGCAACTGGACCAGCCCGAAAACCAGCCCGAGCCATAAAGAACTGTGGACCGGCGCGCAGAGCTTTCTGAGCACCGGCACCACCAACAACCTGAGCGATGATATTAGCAACTATAGCTATGTGGAAGTGTATACCACCCATAAAACCACCGAAAAAACCAAAGGCAACGATAACACCGGCACCATTTGCCATAAATTTTATCTGGATGGCAGCGGCACCTATGTGTGCAGCGGCACCTTTGTGAGCGGCGATCGCACCGATACCAAACCGCCGATTACCGAATTTTATCGCGTGGGCGTGAGCTTTAAAGGCAGCACCTGGACCCTGGTGGATAGCGCGGTGCAGAACAGCAAAACCCAGTATGTGACCCGCATTATTGGCATTAACATGCCG |

**Table S2**: Amino acid sequence of gRBP_Sb1_.

| Protein | | Amino Acid Sequence |
| --- | --- | --- |
| gRBP_Sb1_ | MCGSSHHHHHHSQDPMSKGEELFTGVVPILVELDGDVNGHKFSVRGEGEGDATNGKLTLKFICTTGKLPVPWPTLVTTLTYGVQCFSRYPDHMKRHDFFKSAMPEGYVQERTISFKDDGTYKTRAEVKFEGDTLVNRIELKGIDFKEDGNILGHKLEYNFNSHNVYITADKQKNGIKANFKIRHNVEDGSVQLADHYQQNTPIGDGPVLLPDNHYLSTQSVLSKDPNEKRDHMVLLEFVTAAGITHGMDELYKGSGGGGS**MALNFTTITENNVIRDLTTQVNNIGEELTKERNIFDITDDLVYNFNKSQKIKLTDDKGLTKSYGNITALRDIKEPGYYYIGARTLATLLDRPDMESLDVVLHVVPLDTSSKVVQHLYTLSTNNNQIKMLYRFVSGNSSSEWQFIQGLPSNKNAVISGTNILDIASPGVYFVMGMTGGMPSGVSSGFLDLSVDANDNRLARLTDAETGKEYTSIKKPTGTYTAWKKEFEPKDMEKYLLSSIRDDGSASFPLLVYTSDSKTFQQAIIDHIDRTGQTTFTFYVQGGVSGSPMSNSCRGLFMSDTPNTSSLHGVYNAIGTDGRNVTGSVVGSNWTSPKTSPSHKELWTGAQSFLSTGTTNNLSDDISNYSYVEVYTTHKTTEKTKGNDNTGTICHKFYLDGSGTYVCSGTFVSGDRTDTKPPITEFYRVGVSFKGSTWTLVDSAVQNSKTQYVTRIIGINMP** | |

**Table S3**: Stains used in this study.

| Organism | Characteristics | Source |
| --- | --- | --- |
| *E. coli* | BL21(DE3), protein expression. | Thermo Fisher Scientific |
| *E. coli* | TOP10, plasmid construction, plasmid maintenance. | Thermo Fisher Scientific |
| *S. aureus* | ATCC25923 | ATCC |
| *S. aureus* | ATCC43300, methicillin-resistant. | ATCC |
| *S. aureus* | ATCC49523 | ATCC |
| *S. aureus* | ATCC12600 | ATCC |
| *S. aureus* | LMG10147 | BCCM |
| *S. aureus* | Clinic① | Lab collection |
| *S. aureus* | Clinic② | Lab collection |
| *S. aureus* | Clinic④ | Lab collection |
| *S. aureus* | Clinic⑤ | Lab collection |
| *S. aureus* | Clinic 189 | Lab collection |
| *S. aureus* | Clinic 213, methicillin-resistant. | Lab collection |
| *S. aureus* | Clinic 220 | Lab collection |
| *E. coli* | ATCC25922 | ATCC |
| *K. pneumoniae* | ATCC700603 | ATCC |
| *E. faecium* | LMG16003 | BCCM |
| *A. baumannii* | ATCC17978 | ATCC |
| *P. aeruginosa* PAO1 | ATCC15692 | ATCC |

**Table S4**: Particle sizes and zeta-potentials of the synthesized nanomedicines (n=3).

| Particle | Z-average (d.nm) | Zeta-Potential (mV) |
| --- | --- | --- |
| Nisin-OCT | 189.4±11.4 | 1.2±0.9 |
| Nisin-OCT@RBP_Sb1_ | 255.4±23.0 | -0.8±2.5 |
| Nisin-PA | 138.7±3.5 | 8.1±0.5 |
| Nisin-PA@RBP_Sb1_ | 148.3±2.8 | 0.4±0.1 |
| Nisin@UPSN | 157.5±5.8 | 24.8±0.3 |
| Nisin@UPSN@RBP_Sb1_ | 165.9±4.8 | 14.9±0.5 |

**Table S5**: Protein contents of bacteriophage-mimicking nanomedicines (n = 3).

| Particle | Protein content (nmol/mg) |
| --- | --- |
| Nisin-OCT@RBP_Sb1_ | 1.19±0.13 |
| Nisin-PA@RBP_Sb1_ | 1.32±0.12 |
| Nisin@UPSN@RBP_Sb1_ | 0.86±0.08 |

**Table S6**: Nisin loading efficiency and encapsulation efficiency by wt.% (n = 3).

| Particle | Encapsulation efficiency (%) | Loading efficiency (%) |
| --- | --- | --- |
| Nisin-OCT | 55.3±2.1 | 81.9±5.7 |
| Nisin-OCT@RBP_Sb1_ | 52.6±0.6 | 76.8±0.8 |
| Nisin-PA | 72.1±1.5 | 82.0±1.2 |
| Nisin-PA@RBP_Sb1_ | 73.7±1.4 | 82.8±0.2 |
| Nisin@UPSN | 62.7±1.3 | 64.0±0.4 |
| Nisin@UPSN@RBP_Sb1_ | 73.4±1.2 | 63.0±1.0 |

**Table S7**: Mortality rate of mouse after treatment with Nisin, Nisin-OCT@RBP_Sb1_, Nisin-PA@RBP_Sb1_, or Nisin@UPSN@RBP_Sb1_ (n=10).

| Particle | Mortality rate (%) | | | | | |
| --- | --- | --- | --- | --- | --- | --- |
|  | 0 mg/kg | 32 mg/kg | | 64 mg/kg | 128 mg/kg | 256 mg/kg |
| Nisin | 0 | | 0 | 10 | 40 | 90 |
| Nisin-OCT@RBP_Sb1_ | 0 | | 0 | 0 | 0 | 0 |
| Nisin-PA@RBP_Sb1_ | 0 | | 0 | 0 | 0 | 0 |
| Nisin@UPSN@RBP_Sb1_ | 0 | | 0 | 0 | 0 | 0 |

**Table S8**: Plasmids used in this study.

| Plasmids | Characteristics and purpose | Source |
| --- | --- | --- |
| *pRSF-Cys-His6-GFP-RBP_p545_* | *pRSF-Cys-His6-GFP-RBP_p545_*, encoding Cys-His6-GFP-RBP_p545_ (gRBP_p545_), template. | X. Zhao et al. *Nat. Commun.* 2024, 15, 5287.  Lab collection |
| *pRSF-Cys-His6-GFP-RBP_Sb1_* | *pRSF-Cys-His6-GFP-RBP_p545_* derivative, has a Cys at the N-terminus of the fused protein, expression Cys-His6-GFP-RBP_Sb1_ (gRBP_Sb1_). | This work |

**Table S9**: Primers used in this study.

| Primers | Nucleic acid sequences (5' to 3') | Purpose |
| --- | --- | --- |
| His6-GFP-rv | CGAACCACCTCCTCCACTACCCTTATAAAGCTCATCCATGCCGTG | Amplification of pRSF-Cys-His6-GFP backbond for inserting of RBP_Sb1_ gene. |
| His6-GFP-fw | TAATTAACCTAGGCTGCTGCCACC |  |
| Sb1_RBP-rv | CAGCCTAGGTTAATTACGGCATGTTAATGCCAATAATGC | Amplification of RBP_Sb1_ gene, to generate pRSF-Cys-His6-GFP-RBP_Sb1_.  Sequencing |
| Sb1_RBP-fw | GTGGAGGAGGTGGTTCGATGGCGCTGAACTTTACCAC |  |
| PRSF-T72-Seq-rv | GGTCGTTAAATAGCCGCTTATGTC | Sequencing |
